# Hippocampal versus cortical deletion of cholinergic receptor muscarinic 1 in mice differentially affects post-translational modifications and supramolecular assembly of respiratory chain-associated proteins, mitochondrial ultrastructure, and respiration: implications in Alzheimer’s disease

**DOI:** 10.3389/fcell.2023.1179252

**Published:** 2023-05-24

**Authors:** Mohammad Golam Sabbir, Mamiko Swanson, Robert C. Speth, Benedict C. Albensi

**Affiliations:** ^1^ Division of Neurodegenerative Disorders, St. Boniface Hospital Albrechtsen Research Centre, Winnipeg, MB, Canada; ^2^ Alzo Biosciences Inc., San Diego, CA, United States; ^3^ Canadian Centre for Agri-Food Research in Health and Medicine, St. Boniface Hospital Albrechtsen Research Centre, Winnipeg, MB, Canada; ^4^ Barry & Judy Silverman College of Pharmacy, Nova Southeastern University, Fort Lauderdale, FL, United States; ^5^ Department of Pharmacology and Physiology, School of Medicine, Georgetown University, Washington, DC, United States; ^6^ Department of Pharmacology and Therapeutics, University of Manitoba, Winnipeg, MB, Canada

**Keywords:** muscarinic acetylcholine type 1 receptor CHRM1, hippocampus, cerebral cortex, mitochondria, respiratory complex assembly, respiration, mitochondrial ultrastructure, ATP syntase

## Abstract

**Introduction:** In a previous retrospective study using postmortem human brain tissues, we demonstrated that loss of Cholinergic Receptor Muscarinic 1 (CHRM1) in the temporal cortex of a subset of Alzheimer’s patients was associated with poor survival, whereas similar loss in the hippocampus showed no such association. Mitochondrial dysfunction underlies Alzheimer’s pathogenesis. Therefore, to investigate the mechanistic basis of our findings, we evaluated cortical mitochondrial phenotypes in Chrm1 knockout (Chrm1^−/−^) mice. Cortical Chrm1 loss resulted in reduced respiration, reduced supramolecular assembly of respiratory protein complexes, and caused mitochondrial ultrastructural abnormalities. These mouse-based findings mechanistically linked cortical CHRM1 loss with poor survival of Alzheimer’s patients. However, evaluation of the effect of Chrm1 loss on mouse hippocampal mitochondrial characteristics is necessary to fully understand our retrospective human tissue-based observations. This is the objective of this study.

**Methods:** Enriched hippocampal and cortical mitochondrial fractions (EHMFs/ECMFs, respectively) derived from wild-type and Chrm1^−/−^ mice were used to measure respiration by quantifying real-time oxygen consumption, supramolecular assembly of oxidative phosphorylation (OXPHOS)-associated proteins by blue native polyacrylamide gel electrophoresis, post-translational modifications (PTMs) by isoelectric focusing (IEF), and mitochondrial ultrastructure by electron microscopy.

**Results:** In contrast to our previous observations in Chrm1^−/−^ ECMFs, EHMFs of Chrm1^−/−^ mice significantly increased respiration with a concomitant increase in the supramolecular assembly of OXPHOS-associated proteins, specifically Atp5a and Uqcrc2, with no mitochondrial ultrastructural alterations. IEF of ECMFs and EHMFs from Chrm1^−/−^ mice showed a decrease and an increase, respectively in a negatively charged (pH∼3) fraction of Atp5a relative to the wild-type mice, with a corresponding decrease or increase in the supramolecular assembly of Atp5a and respiration indicating a tissue-specific signaling effect.

**Discussion:** Our findings indicate that loss of Chrm1 in the cortex causes structural, and physiological alterations to mitochondria that compromise neuronal function, whereas Chrm1 loss in the hippocampus may benefit neuronal function by enhancing mitochondrial function. This brain region-specific differential effect of Chrm1 deletion on mitochondrial function supports our human brain region-based findings and Chrm1^−/−^ mouse behavioral phenotypes. Furthermore, our study indicates that Chrm1-mediated brain region-specific differential PTMs of Atp5a may alter complex-V supramolecular assembly which in turn regulates mitochondrial structure-function.

## 1 Introduction

Cholinergic neurons communicate using the neurotransmitter acetylcholine (ACh). There are two major subtypes of acetylcholine receptors (AChRs); metabotropic muscarinic receptors (mAChRs) and ionotropic nicotinic receptors. The former are G-protein-coupled receptors (GPCRs), whereas the latter are ligand-gated ion channels. AChR-mediated modulation of neuronal activity is critical to normal brain function, i.e., sensory information processing (Minces et al., 2017), attention (Sarter et al., 2005), cognition (Everitt and Robbins, 1997; Sarter and Parikh, 2005), learning and memory (Bartus et al., 1982; Maurer and Williams, 2017), sleep (Xu et al., 2015), and arousal (Szymusiak, 1995). Altered cholinergic receptor expression and function have been described in several neurodegenerative diseases including Alzheimer’s (AD), Parkinson’s (PD), and Huntington’s disease (HD) as well as in psychiatric disorders such as schizophrenia (Tata et al., 2014). Degeneration of cholinergic neurons and cholinergic hypofunction are considered to be major contributors to the pathologies associated with AD (Pavia et al., 1998; Jiang et al., 2014).

The metabotropic mAChRs consist of five subtypes encoded by the CHRM1-CHRM5 genes, that are expressed in a tissue and cell-type-specific manner in non-neuronal peripheral tissues (Wessler and Kirkpatrick, 2008) as well as in neurons and glial cells of the central and peripheral nervous systems (CNS and PNS) (Gong et al., 2003; Eglen, 2005; Wess et al., 2007). Direct stimulation of CHRM1 using agonists and allosteric modulators enhanced cognition in animal models and improved performance in cognitive tests in Alzheimer’s patients (Clader and Wang, 2005; Tarr et al., 2012). Therefore, CHRM1 has been postulated to be an important therapeutic target for neurodegenerative diseases, specifically AD (Jiang et al., 2014). The pathogenic role of CHRM1 in AD is supported by the observation that CHRM1 is abundantly expressed in both cerebral cortex and hippocampus in non-demented individuals (Giraldo et al., 1987; Ehlert and Tran, 1990; Levey, 1996) whereas it is severely downregulated in the majority of AD patients (Sabbir et al., 2022). These brain regions manifest the hallmark neuropathology of AD during the early stage of disease progression (Braak and Braak, 1991; Palmqvist et al., 2017). Both brain regions are associated with learning and memory processes which are impaired in AD. These observations led us to a recent retrospective study involving a large cohort of postmortem human brain tissues, analyzing CHRM1 protein abundance in the hippocampus and temporal cortex of AD patients and age/sex-matched nondemented individuals (Sabbir et al., 2022). Our study revealed a dramatic loss of CHRM1 protein (≥50% decrease) in the temporal cortex and hippocampus of a subset of AD patients (Sabbir et al., 2022). Furthermore, it was found that temporal cortical loss, but not hippocampal loss of CHRM1 protein was significantly associated with poor patient survival (Sabbir et al., 2022). These findings raised an important question: what are the molecular and physiological consequences of CHRM1 loss in the hippocampus versus the cortex that differentially affected the survival of AD patients?

In animal models, a definitive molecular interpretation of the role of CHRM1-regulated behavioral and pathological phenotypes in the development and progression of AD is still lacking. One particular aspect, so far neglected, is the role of CHRM1 in regulating mitochondrial function. Several mediators of the GPCR signal transduction pathway are localized in the mitochondria, for example, different G proteins (Gα_12_, Gα_i_, and Gβγ) (Lyssand and Bajjalieh, 2007; Andreeva et al., 2008; Zhang et al., 2010) and the kinase of the G protein-coupled receptor type 2 (GRK2) (Fusco et al., 2012). Functional involvement of these proteins in regulating mitochondrial physiology is supported by the observation that activation of β2-adrenergic receptors (GPCRs) by catecholamines translocates GRK2 to the mitochondria, reduces ATP loss, and induces mitochondrial biogenesis (Ciccarelli et al., 2013). Interestingly, GRK2 is a CHRM1 desensitizing enzyme (Haga et al., 1996; Yeatman et al., 2014), therefore, it is possible that CHRM1 signaling may also exert similar effects on mitochondrial physiology through GRK2 translocation. On the other hand, CHRM1-G proteins and β-Arrestin signaling activate extracellular signal-regulated kinases (ERK1/2) (Ahn et al., 2004; Goldsmith and Dhanasekaran, 2007) that have been localized to mitochondria (Alonso et al., 2004), directly or indirectly regulating a myriad of mitochondrial functions (Javadov et al., 2014; Cook et al., 2017). Thus, there is compelling evidence of a link between CHRM1 signaling and the regulation of mitochondrial function. However, a direct cause-and-effect relationship has yet to be established.

Neuronal activity is fueled by glycolysis and oxidative phosphorylation (OXPHOS) to produce ATP from circulating glucose (Davis, 2020). It has been calculated that resting cortical neurons consume 4.7 billion ATP molecules per second (Zhu et al., 2012). Also, it has been estimated that over 80% of the energy in myelinated hippocampal axons is expended by postsynaptic potentials (Alle et al., 2009; Harris et al., 2012). Furthermore, presynaptic vesicle recycling acts as an additional energy-consuming process. Therefore, it is not surprising that the disruption of mitochondrial functioning underlies AD pathogenesis (Hirai et al., 2001; Wang et al., 2020; Mary et al., 2022; Reiss et al., 2022). Most cortical neurons express CHRM1 (Mrzljak et al., 1993; Fernández de Sevilla et al., 2021). Therefore, to understand the role of CHRM1 to sustain normal mitochondrial function, mitochondrial pathophysiologies arising from the loss of CHRM1 may inform the mechanisms leading to the progression of AD. Recently, we characterized the molecular biology of cortical mitochondria in Chrm1 deleted (Chrm1^−/−^) transgenic mice (Sabbir et al., 2023). Our findings indicate that Chrm1 loss severely reduced cortical mitochondrial respiration, associated with a significantly decreased supramolecular assembly of OXPHOS-associated protein complexes, specifically the Atp5a subunit of ATP synthase. Additionally, an abnormal cortical mitochondrial ultrastructure was revealed. These are important regulators of mitochondrial function (Sabbir et al., 2023). It has been suggested that long rows of oligomerized ATP synthase dimers facilitate the convexity of the mitochondrial inner membrane at the apex of the cristae shaping its structure and appearance (Nesci and Pagliarani, 2019). The reduced Atp5a oligomerization in Chrm1^−/−^ cortical mitochondria corresponded with abnormal cristae structure in the cortical neuropil synaptic bouton mitochondria, supporting a role for Chrm1 in regulating cristae shape by controlling ATP synthase oligomerization (Sabbir et al., 2023). Our study established a cause-and-effect relationship between Chrm1 signaling loss and mitochondrial structural-functional deficits in cortical neurons (Sabbir et al., 2023). In light of these findings, the objective of this study is to analyze molecular, structural, and physiological characteristics of hippocampal mitochondria in Chrm1^−/−^ and wild-type mice to understand the molecular basis of any brain region-specific effect on mitochondrial phenotypes and to extrapolate those findings to our retrospective human brain region-specific observations to determine why hippocampal CHRM1 loss was not associated with poor survival of Alzheimer’s patient (Sabbir et al., 2022).

## 2 Materials and methods

### 2.1 Chrm1 knockout mouse

Targeted Chrm1 deleted mouse (Chrm1^−/−^) line (Sabbir et al., 2022) was provided by Dr. Jurgen Wess, National Institutes of Health (NIH). All animal procedures followed the guidelines of the University of Manitoba Animal Care Committee using the Canadian Council of Animal Care rules. Six months old adult male mice were used for isolation of the hippocampus. Previously, in our retrospective human tissue-based study, we observed no sex-specific difference between CHRM1 loss and survival of Alzheimer’s patients (Sabbir et al., 2022), therefore, for simplicity, we have not included female mice in our study which may be the subject of future studies. We used a cohort of twenty-five adult (6–7 months old) male Chrm1^−/−^ mice and corresponding age-matched wild-type mice to perform the different experiments in this study.

### 2.2 Postmortem human brain tissue

Frozen (−80°C) postmortem human hippocampus tissue samples were provided to Dr. Sabbir by the NIH NeuroBioBank (Sample request number: 1883).

### 2.3 Isolation and enrichment of the mitochondria

Enriched hippocampal mitochondrial fractions (EHMFs) were prepared from freshly harvested adult male mice brains by a method previously described by Sabbir et al. (2020), Sabbir et al. (2021) and Sabbir et al. (2023). The hippocampus was first diced on ice and added to a mitochondrial isolation buffer (MIB) containing 70 mM sucrose, 210 mM mannitol, 5 mM HEPES pH 7.2, and 1 mM EGTA. The tissues were homogenized with a Teflon Dounce homogenizer, then centrifuged at 800 g for 10 min at 4°C. The supernatant was passed through two layers of cheesecloth into a separate tube and centrifuged at 8,000 g for 10 min at 4°C. This precipitated the enriched mitochondrial fractions as a pellet. The supernatant was discarded, and the pellet was resuspended in the MIB, and centrifuged again. The final pellet was resuspended with a minimal volume of MIB and the total protein concentration was determined. The resuspended freshly harvested EHMFs were used in downstream applications.

### 2.4 Mitochondrial function test

The coupling and electron flow assays were performed using 5 µg of EHMFs using a Seahorse XF24 analyzer (Agilent) following a technique previously standardized using a cortical enriched mitochondrial fraction (ECMFs) as described by Sabbir et al. (2021). For the coupling assay, succinate (10 mM) was used as a substrate in the presence of rotenone (2 µM), while pyruvate (10 mM) and malate (2 mM) were used as substrates in the electron flow assay in the presence of 4 µM carbonyl cyanide-p-trifluoromethoxyphenylhydrazone (FCCP). For the coupling assay, the oxygen consumption rate (OCR) was measured while ADP (4 mM), oligomycin (2.5 μg/mL), FCCP (4 µM), and antimycin A (4 µM) were added sequentially. For the electron flow assay, the OCR was measured while 2 µM rotenone, 10 mM succinate, 4 µM antimycin A, 10 mM ascorbate +100 µM (N, N, N9, N9-Tetramethylp-phenylenediamine; TMPD) were injected sequentially.

### 2.5 Blue-native polyacrylamide gel electrophoresis (BN-PAGE)

Two-dimensional (2D) BN-PAGE/SDS-PAGE analysis was performed as described by Sabbir et al. (2021) (Sabbir, 2018; Sabbir, 2019). The EHMFs and human brain tissues (hippocampus) were lysed in a buffer containing 1X phosphate-buffered Saline (PBS), 1X Halt protease and phosphatase inhibitor cocktail (catalog number: 1861281, Thermo Scientific), and 1.5% n-Dodecyl β-D-maltoside (catalog number: D4641, Sigma) which was then sonicated. A 4%–15% gradient BN-PAGE gel was used to separate the native proteins. The individual lanes were cleaved into gel slices and submerged in the Laemmli sample buffer consisting of 100 mg/mL of dithiothreitol (DTT) for 30 min at room temperature. The proteins for each lane were resolved in the second dimension SDS-PAGE and immunoblotted.

### 2.6 Transmission electron microscopy (TEM)

The mice were perfused with formaldehyde for 15 min and dissected to acquire the hippocampal tissue. The tissues were then fixed in glutaraldehyde for 2 h, then postfixed in osmium tetroxide for an hour. The samples were washed three times with distilled water for 5 min each, then passed through alcohol gradients to dehydrate the tissues followed by transitional dehydration in propylene oxide for 2 min × 15 min. The tissue samples were treated with propylene oxide for an hour and finally infiltrated by full plastic overnight at 70°C. To determine the orientation of the tissues, semithin (500 nm) sections were cut. A Leica ultramicrotome (Leica EM UCF7) was used to cut ultrathin sections (<90 nm) which were mounted on a copper grid (200 square mesh, Cat: V2200, Canemco Inc.). The sections were stained with uranyl acetate and lead citrate, then imaged using a JEOL transmission electron microscope (model: JEOL JEM-1010, JEOL USA Inc.) with AMT image capture engine software version V602.5.

### 2.7 Western blotting (WB) and immune-detection

Relative quantification based on WB of proteins was previously described in detail by Sabbir et al. (2020). A cocktail of 5 antibodies (Table 1) specific to different subunits of human or rodent OXPHOS complexes, namely, Ndufb8 (complex-I), Sdhb (complex-II), Uqcrc2 (complex-III), Mtco1/2 (complex-CIV), and Atp5a (complex-V) were used to determine the relative abundances of complexes-I-V (Table 1).

**TABLE 1:** List of antibodies.

Antibody	Catalog number	Vendor	Type	Lot number
Anti-CHRM1	Sc-365966 (G-9)	SCBT	Monoclonal	F2812
Anti-VDAC1	Ab14734	Abcam	Monoclonal	GR243577-4
Anti-OXPHOS-rodent containing a cocktail of Anti-NDUFB8, Anti-SDHB, Anti-UQCRC2, Anti-MT-CO1, and Anti-ATP5A antibodies	MS604-300 (ab110242, ab14714, ab14745, ab14705, and ab14748, respectively)	Abcam	Monoclonal	K2342
OXPHOS-human containing a cocktail of Anti-NDUFB8, Anti-SDHB, Anti-UQCRC2, Anti-MT-CO2, and Anti-ATP5A antibodies.	Ab110411 (ab110242, ab14714, ab14745, ab110258, and ab14748, respectively)	Abcam	Monoclonal	R3362
Anti-ATP5B	MS503	MitoScience	Monoclonal	B0827

### 2.8 Isoelectric focusing (IEF)

Isoelectric focusing of the protein was performed as described previously by Sabbir (Sabbir et al., 2022). Briefly, 50 μg of total cell lysate from the EHMF and ECMFs were precipitated by acetone and dissolved in a rehydration buffer containing 8 M Urea, 2% CHAPS, 50 mM dithiothreitol (DTT) and 0.2% Bio-Lyte ampholytes pH3-10. The dissolved proteins were then incubated in immobilized pH gradient (IPG) strips (NL) (Bio-Rad) for 1 h and focused at 175 volts (V) for 15 min, 175–2,000 V ramp for 45 min, and 2,000 V for 30 min. After focusing, the proteins in the strips were reduced using DTT, alkylated using iodoacetamide, resolved by SDS-PAGE, and immunoblotted.

### 2.9 Statistical analysis

Prism version 7.00 (GraphPad Software) was used to perform statistical analysis as described previously (Sabbir et al., 2018; Sabbir et al., 2020; Sabbir et al., 2021; Sabbir et al., 2022). The means of the wild-type and Chrm1^−/−^ groups were compared by a two-tailed unpaired *t*-test. A two-way ANOVA test was performed to analyze the effect of Chrm1 deletion and brain region (hippocampus versus cortex)-specificity on the PTM status of different OXPHOS-associated proteins. A Pearson correlation coefficient (two-tailed) was computed to assess the linear relationship between the relative proportion of post-translationally modified fractions of OXPHOS-associated proteins in ECMFs and EHMFs under Chrm1 deletion condition. Differences were considered significant with *p* < 0.05 and throughout the text, if a *p*-value is ≤0.05, ≤0.01, ≤0.001, or ≤0.0001, it was flagged with one, two, three, or four asterisks, respectively.

## 3 Results

### 3.1 A 72 kilodalton (kDa) Chrm1 protein is expressed in the hippocampus

**FIGURE 1:**
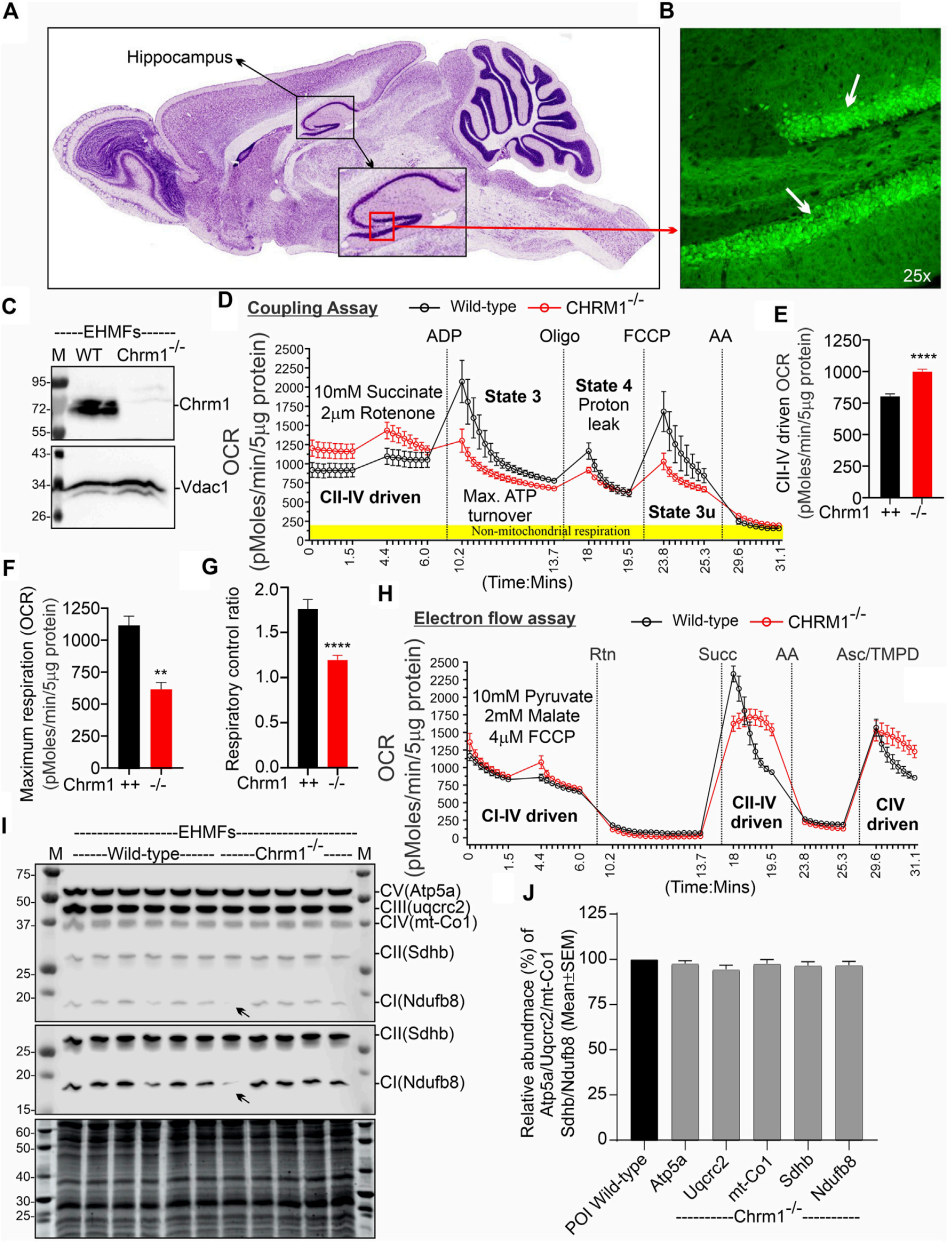
Chrm1 protein is abundant in mouse hippocampus and the loss of Chrm1 affected respiration in EHMFs. **(A, B)** A representative image **(A)** showing cresyl violet-stained sagittal section of the GENSAT BAC transgenic Chrm1 enhanced green fluorescence (eGFP) reporter mouse brain highlighting the hippocampal region (black dotted circle). The black square indicates the dentate gyrus (DG) region. A magnified representative immunofluorescence image of the DG is shown in panel B demonstrating the abundance of eGFP corresponding to native Chrm1 expression in the hippocampal pyramidal neurons (white arrows). **(C)** Immunoblots showing the relative abundance of Chrm1 and Vdac1 in EHMFs derived from wild-type and Chrm1^−/−^ mice. **(D, H)** Line graphs showing OCR kinetics in coupling **(D)** and electron flow **(H)** assays performed simultaneously in a Seahorse 24X flux analyzer using 5 µg protein equivalent of EHMFs from adult wild-type and Chrm1^−/−^ mice. The final concentration of the inhibitors and substrates is mentioned in the text. The data were generated using a “point-to-point” mode in the Seahorse XF24 software package. The point-to-point displays OCR as a series of rates across the measurement period and can show changes in the rate across the measurement period. The OCR kinetics data presented in E and G was converted to a “middle point” mode using Seahorse 24X flux analyzer software utility which is a preferred method for statistical comparison between wild-type and chrm1^−/−^. The middle point mode shows a single OCR value for the measurement period which is the average of the point-to-point rates. **(E)** Bar graph showing basal respiration (coupled) driven by CII-IV. N = Three adult male mice in each group with 6–7 replicates per animal. Data represented as Mean ± SEM. Comparison of the means involving wild-type and Chrm1^−/−^ groups in this dataset and subsequent datasets were by *t*-test (unpaired). *p* < 0.0001. This dataset was used to calculate the maximal respiration and respiratory control ratio (RCR) in the subsequent figures. **(F)** Bar graph showing maximum respiration under state 3. The non-mitochondrial respiration was subtracted to calculate maximum respiration. *p* = 0.0016. **(G)** Bar graph showing the RCR (State 3/State 4). Data represented as mean ± SEM. *p* = 0.0007. **(I)** Representative immunoblots showing the relative abundance of OXPHOS proteins (Atp5a, uqcrc2, mt-Co1, Sdhb, and Ndufb8) with the Oriole-stained representative total protein gel below. **(J)** Bar graph showing the relative amount (%; mean ± SEM) of OXPHOS proteins in EHMFs. The normalization of the protein of interest (POI) was performed based on total protein loading (oriole-stained gel) (Sabbir et al., 2020) and has been represented as a percentage of wild-type protein level (100%). N = 5 wild-type and 5 Chrm1^−/−^ adult mice with 4 replicates per animal.

A Chrm1 promoter-trapped enhanced green fluorescence protein (eGFP) reporter mouse-based study under the Gene Expression Nervous System Atlas (GENSAT) project revealed that Chrm1 is expressed in mouse hippocampal pyramidal neurons (Tomishima et al., 2007; Schmidt et al., 2013) (Figures 1A, B). Therefore, to study the effect of Chrm1 loss in hippocampal mitochondrial structure and function, we harvested EHMFs using a differential centrifugation technique that was extensively validated in our previous studies (Sabbir, 2019; Sabbir et al., 2021; Sabbir et al., 2023) for effectiveness in isolation and enrichment of ECMFs from mouse brain (Sabbir et al., 2023). TEM images of the EHMF revealed the presence of free isolated mitochondria (Supplementary Figures S1A, B, blue arrows) as well as pre- (electron-dense and potential neurotransmitter containing vesicles, Supplementary Figure S1B, green arrows) and postsynaptic (relatively less electron-dense with few vesicular structures, Supplementary Figure S1B, orange arrows) dendritic membrane-enclosed mitochondria (Supplementary Figure S1B, pink and yellow arrows, respectively). Immunoblotting using a validated anti-Chrm1 antibody (Table 1) and Chrm1 knockout mouse tissues revealed the presence of ∼72 kDa Chrm1 in the EHMFs (Figure 1C) as has been previously observed in the ECMFs (Sabbir et al., 2023). The theoretical molecular weight of mouse Chrm1 protein (Transcript Id: ENSMUST00000035444.9) is 51 kilodaltons (kDa). Previously, we demonstrated that Chrm1 appears as 72 kDa forms in Western blotting due to post-translational modifications (PTMs), specifically glycosylation (Sabbir et al., 2022).

### 3.2 Loss of Chrm1 significantly improved complex II-IV-driven respiration in isolated hippocampal mitochondria under a coupled state

We performed coupling and electron flow assays (Sabbir et al., 2021) to assess isolated hippocampal mitochondrial function under Chrm1 deletion condition. The working principles of coupling and electron flow assays have been previously described in detail by Sabbir et al. (2021). The coupling assay measures the level of respiratory coupling between the electron transport chain (ETC) and the oxidative phosphorylation (OXPHOS) system which is reflected by the increase in respiration in response to ADP (Figure 1D), whereas the electron flow assay is designed to follow and interrogate each complex of the electron transport system under an uncoupled state by the addition of FCCP (Figure 1H). In the coupling assay, basal respiration was measured in terms of oxygen consumption rate (OCR) when succinate was supplied as substrate, and complex I was inhibited by rotenone (Figure 1D). The complex II-IV mediated respiration was significantly (*p* < 0.0001) increased in Chrm1^−/−^ EHMFs compared to wild-type (Figure 1E). This finding is in contrast to our previous finding in cortical tissues where Chrm1 loss decreased basal coupled respiration in ECMFs compared to wild-type (Table 2). The addition of ADP increased OCR as expected (state 3 respiration), however, Chrm1^−/−^ EHMFs exhibited decreased OCR compared to wild-type (Figure 1D). This caused significantly decreased (*p* = 0.0013) maximal respiratory capacity in Chrm1^−/−^ EHMFs compared to wild-type (Figure 1F). The drop in the maximal respiratory capacity in EHMFs showed equivalence to a similar effect in the ECMFs which is summarized in Table 2.

**TABLE 2:** Differential effect of Chrm1 loss on multiple molecular and physiological aspects of cortical and hippocampal mitochondria.

Molecular/physiological/ultrastructural phenotypes	Cortex	Hippocampus
Coupled respiration		
Complex II-IV driven respiration	**Decreased**	**Increased**
State 3	Decreased	Decreased
State 4	Decreased	Unaltered
Uncoupled Respiration (+FCCP)		
Complex I-IV driven respiration	Decreased	Unaltered
Complex II-IV driven respiration	Decreased	Unaltered
Complex IV driven respiration	Decreased	Unaltered
OXPHOS protein abundance	Unaltered	Unaltered
Megacomplex (≥720 kDa)		
Atp5a (Complex V)	**Decreased**	**Increased**
mt-Co1 (Complex IV)	Decreased	**Increased**
Uqcrc2 (Complex III)	Decreased	**Increased**
Sdhb (Complex II)	Decreased	**Increased**
Ndufb8 (Complex I)	Decreased	Unaltered
Supercomplex (100–500 kDa)		
Atp5a (Complex V)	Unaltered	Unaltered
mt-Co1 (Complex IV)	Unaltered	Unaltered
Uqcrc2 (Complex III)	Unaltered	Unaltered
Sdhb (CII)	Decreased	Unaltered
Ndufb8 (CI)	Decreased	Unaltered
PTMs: negatively charged fraction, potential phosphorylation		
Atp5a (CV)	**Decreased**	**Increased**
mt-Co1 (CIV)	**Increased**	**Increased**
Sdhb (CII)	Altered*	Unaltered
Mitochondrial ultrastructure in the neuropil		
Abnormal cristae	**Yes**	**No**
Tinctorial property of pyramidal neurons		
Dark neuron abundance	Increased	Unaltered
Endoplasmic reticulum in dark neuron	Enlarged	Normal
Nature of synapse	Altered#	Unaltered

Asterisk: alteration in the negative and positive charged fractions (Figures 3F, I pink arrow and yellow dotted rectangle). #: alteration in the appearance of the tinctorial property (less electron-dense based on the uranium and lead staining) post-synaptic dendritic process.

Bold words hightlight the contrasting effects in the mitochondrial phenotypes under Chrm1 deletion condition in the cortex versus hippocampus.

The addition of the ATP synthase inhibitor oligomycin decreased OCR in both Chrm1^−/−^ and wild-type EHMFs. Following oligomycin addition, the observed residual OCR was due to a proton leak (Figure 1D). The mitochondrial proton leak is measured in terms of OCR under non-phosphorylating conditions (Porter et al., 1999; Jastroch et al., 2010). We measured the coupling efficiency (the percentage of respiration rate at a given mitochondrial membrane potential that is used for ATP synthesis) by calculating the respiratory control ratio (RCR: State 3/State 4) (Sabbir et al., 2021). The RCR was significantly (*p* < 0.0001, unpaired *t*-test) decreased in the Chrm1^−/−^ EHMFs compared to wild-type (Figure 1G). A high RCR means efficient and healthy mitochondrial functioning because it implies that the mitochondria have a high capacity for substrate oxidation and a low proton leak (Brand and Nicholls, 2011). We anticipated that Chrm1^−/−^ EHMFs would exhibit a high RCR compared to the wild-type because the basal respiration was significantly higher in this condition compared to the wild-type (Figure 1E). In contrast, the relatively low state 3 respiration (Figure 1F) and unaltered (not statistically different, *p* > 0.05) proton leak (Figure 1D) under Chrm1 loss condition compared to the wild-type caused a significant (*p* = 0.0007) decrease in the RCR in Chrm1^−/−^ EHMFs compared to wild-type. In this instance, the drop in the RCR in Chrm1^−/−^ EHMFs may not be a true reflection of mitochondrial functionality. In the existing literature, RCR is used as a measure of mitochondrial functionality, but its value depends on numerous factors and may change in a fluctuation of almost any aspect of OXPHOS (Brand and Nicholls, 2011). Overall, the coupling assay revealed significantly increased mitochondrial OXPHOS functioning under a coupled state in Chrm1^−/−^ EHMFs compared to wild-type.

The electron flow assay revealed that the complexes I–IV (pyruvate and malate as substrate), complexes II–IV (in presence of complex-1 inhibitor rotenone and succinate as substrate), and complex IV (in presence of complex III inhibitor antimycin A and ASC/TMPD as substrate)-mediated uncoupled state (in the presence of uncoupler FCCP) respiration was relatively unaltered (statistically not significant, *p* > 0.05) in Chrm1^−/−^ EHMFs compared to wild-type (Figure 1H). This is in contrast to our previous findings in a similar assay using ECMFs where the loss of Chrm1 significantly decreased complexes 1–IV/complexes II–VI/complex IV driven uncoupled state respiration compared to wild-type (Table 2) (Sabbir et al., 2023). Overall, these findings indicate an inverse effect on both coupled and uncoupled states of respiration in cortical versus hippocampal mitochondria under Chrm1 loss condition. Such tissue-specific contrasting effect of Chrm1 on mitochondrial physiology was not reported previously (see Table 2 for a summary of the findings).

### 3.3 Loss of Chrm1 in the hippocampus exhibited no effect on the relative abundance of key OXPHOS-associated proteins

We examined the relative abundance of NADH: Ubiquinone Oxidoreductase Subunit B8 (Ndufb8), Succinate Dehydrogenase Complex Iron-Sulfur Subunit B (Sdhb), Ubiquinol-Cytochrome C Reductase Core Protein 2 (Uqcrc2), Mitochondrially Encoded Cytochrome C Oxidase I (mt-Co1), and ATP Synthase F1 Subunit Alpha (Atp5a) protein subunits of complex I, complex II, complex III, complex IV, and complex V respectively, representing different respiratory complexes by Western blotting using a cocktail of antibodies. This is to explore the possibility of any alteration in the abundance of these protein subunits representing the relative abundance of respiratory complexes as an underlying factor mechanistically explaining the basal respiratory improvement in Chrm1^−/−^ EHMFs. Quantification based on immunoblotting (Figure 1I) revealed no statistical difference (*p* > 0.05) in the relative amount of these proteins in the EHMFs under Chrm1 loss condition compared to wild-type (Figure 1J).

### 3.4 Chrm1 loss in the hippocampus significantly improved the supramolecular organization of mitochondrial ATP synthase and complex II–IV-associated respiratory megacomplexes

The current plasticity model of the structural organization of mitochondrial respiratory complexes suggests that instead of being independently moving entities connected by mobile electron carriers Coenzyme Q and cytochrome c, complex 1–IV are assembled into supramolecular structures known as Supercomplexes (SCs: containing complex I, complex III, and complex IV in different proportions) (Enríquez, 2016) or Megacomplexes (MCs: larger association of SCs, for example, a row-like organization of SCs or a string of dimeric ATP synthases) (Bultema et al., 2009). It has been suggested that the supramolecular respiratory structures underlie the increased efficiency of the ETC, and reduces the rate of reactive oxygen species (ROS) production (Novack et al., 2020). The majority of the complex-II in mammalian mitochondria are present as free non-associated stand-alone forms, while only a small proportion is associated with SCs (Kovářová et al., 2013; Vartak et al., 2013). The respiratory SC/MC assemblies represent the highest-order assembly of OXPHOS complexes allowing mitochondria to respond to energy-requiring conditions. Previously, we demonstrated that loss of Chrm1 in mouse cortex led to a dramatic reduction in the ultrastructural organization of ATP Synthase (represented by the oligomerization of Atp5a) and relatively reduced supramolecular assembly of complexes I–IV-associated SC/MC structures, both factors were associated with respiratory deficits in the Chrm1^−/−^ ECMFs compared to wild-type (Table 2) (Sabbir et al., 2023). Therefore, to understand the molecular basis of enhanced respiration in Chrm1^−/−^ EHMFs compared to wild-type, we studied SC/MC assembly by 2D BN-PAGE/SDS-PAGE followed by immunoblotting using anti-OXPHOS cocktail antibodies.

**FIGURE 2:**
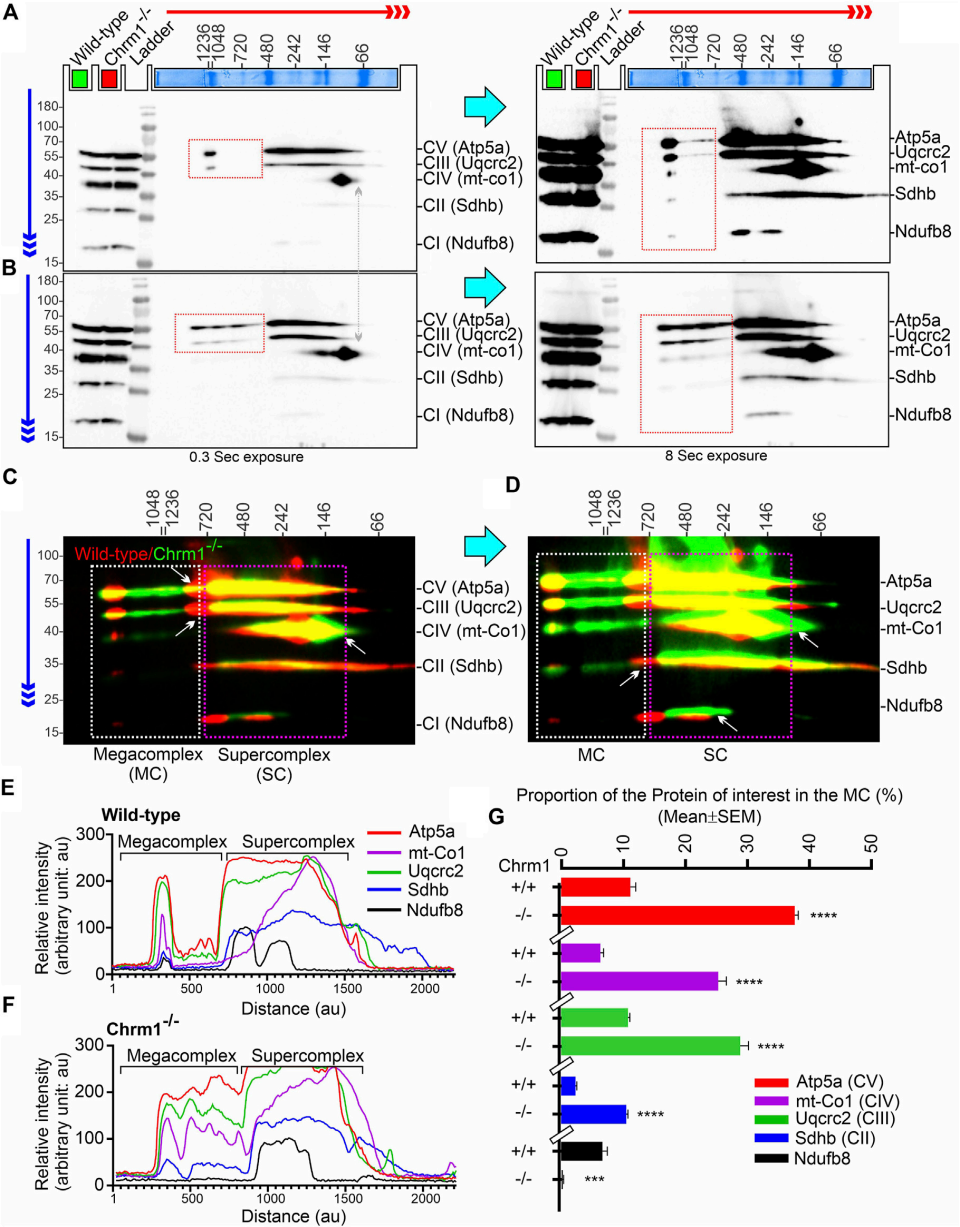
2D BN-PAGE/SD-PAGE analysis revealed that loss of Chrm1 led to an increased supramolecular assembly of respiratory protein complexes in EHMFs. **(A, B)** Immunoblots showing OXPHOS-associated MPCs in wild-type **(A)** and Chrm1^−/−^ **(B)** EHMFs. The immunoblot was generated by the simultaneous use of a cocktail of five antibodies (Table 1). Representative total protein lysates were loaded in two left lanes (green and red colored rectangles) in the 2D SDS-PAGE to highlight that the relative amount of OXPHOS proteins was not altered but their supramolecular assembly was altered under Chrm1 loss condition. The red and blue arrows indicate the direction of electrophoresis during 1D BN-PAGE and 2D SDS-PAGE respectively. The red dotted rectangle indicates ≥∼720 kDa Atp5a and Uqcrc2 associated MCs. **(C, D)** Immunoblots in A and B were false-colored and overlaid to show relative abundance/shift of OXPHOS-associated MCs (white dotted rectangle, ≥720 kDa) and SCs (pink dotted rectangles, 720–100 kDa). Vertical alignment of OXPHOS-associated proteins indicates a potential association between MPCs that co-migrated during 1D BN-PAGE. **(E, F)** Image J-based plot profile of the immunoblots presented in A and C (higher exposures). **(G)** Bar graph showing the relative abundance of Atp5a (complex V), mt-Co1 (complex IV) Uqcrc2 (complex III), Sdhb (complex II), and Nsufb8 (complex I) subunits potentially recruited as MCs as they co-migrated (aligned vertically) in 1D BN-PAGE. Data presented as Mean ± SEM. N = 5 independent experiments using 5 adult male mice in each group. In all datasets, the means of wild-type and Chrm1^−/−^ groups were compared by *t*-test (unpaired). The rectangular bars placed on the X-axis indicate the grouping of the datasets by a discontinuous X-axis. The same strategy is used for grouping datasets in the remaining figures. Atp5a,: Mt-Co1, Uqcrc2, and Sdhb: *p* < 0.0001, ndufb8: *p* = 0.0002.

The supramolecular assembly of the OXPHOS MPCs exhibited a considerable difference between Chmr1^−/−^ and wild-type EHMFs (Figure 2). The Atp5a (complex-V) appeared as monomeric to dimeric to oligomeric complexes ranging from ∼66 kDa to ≥ 1 Megadalton (MDa) MPCs (Figures 2A–D). Quantification based on immunoblots and plot profiles of the protein complexes revealed significantly increased ≥720 kDa Atp5a oligomers (Figures 2A–F) in Chmr1^−/−^ EHMFs compared to wild-type (Figure 2G). The molecular weight of the human mitochondrial respiratory chain SC (SC: complexes I_1_III_2_IV_1_) has been reported as ≥ 1.7 MDa (Guo et al., 2017a). False-colored overlapped immunoblots of the OXPHOS MPCs profiles revealed vertical alignment of Ndufb8, Sdhb, Uqcrc2, and mt-Co1 oligomers in the ≥ 720 kDa and 100–720 kDa regions (Figures 2C, D, white and pink dotted rectangles, respectively). Based on the reports in existing literature (Acín-Pérez et al., 2008), we considered ≥ 720 kDa and 100–720 kDa MPCs as MCs and SCs, respectively (Figures 2E, F). Quantification based on immunoblots (Figures 2A, B) and plot profiles (Figures 2E, F) revealed a significantly increased proportion of ≥ 720 kDa complexes II–IV MCs in Chmr1^−/−^ EHMFs compared to wild-type (Figure 2G). On the other hand, the appearance of the 100–720 kDa Atp5a, Uqcrc2, mt-Co1, Sdhb, and Ndufb8-associated SCs was not affected under Chrm1 loss condition (Figures 2C, D, pink dotted rectangle). However, it is noticeable that the complexes I–V SCs in Chmr1^−/−^ EHMFs shifted to a relatively lower molecular weight region compared to wild-type (Figure 2C, white arrows) which is possibly due to an increased proportion of the corresponding protein subunits recruited to the MCs causing this shift in the proportion of SCs. Overall findings indicate loss of Chrm1 led to a dramatic increase in the ultrastructural organization of ATP Synthase (Atp5a) and complexes II-IV-associated MCs which may underlie the observed enhancement in complexes II-IV-mediated respiration in the EHMFs.

### 3.5 Comparison of cortex versus hippocampus revealed Chrm1 loss differentially altered post-translational modifications (charged fractions) of Atp5a and Uqcrc2 proteins

We performed IEF of the OXPHOS proteins, specifically Atp5a and Uqcrc2, the two subunits of complex V and complex III, respectively that exhibited a dramatic alteration in the supramolecular organization under Chrm1 loss condition (Figures 2A, B) which correlated to altered respiration (Figure 1D). It has been suggested that PTMs, specifically phosphorylation can alter the supramolecular organization of proteins. For example, mammalian small heat shock proteins (HSPs) rapidly phosphorylate and oligomerize to form 200–700 kDa and > 5,000 kDa aggregates in response to stress and mitogenic signals (Benndorf et al., 1994). It has been demonstrated that the degree of HSP25 protein phosphorylation determines its structural organization (polymerization) which in turn affects its actin polymerization-inhibiting activity (Benndorf et al., 1994). Therefore, we expected to see a difference in the charged fractions (potentially phosphorylated) of Atp5a and Uqcrc2 correlating to their tissue-specific (hippocampus versus cortex) differential supramolecular organization and respiratory function (Table 2). Phosphorylation of Atp5a and Uqcrc2 has not yet been characterized and there are no phospho-epitope-specific antibodies commercially available. Therefore, IEF is a convenient method of our choice to study PTMs of these proteins. IEF separates proteins based on their isoelectric point (pI) and can readily detect phosphorylated forms as each additional phosphate causes a negative shift in pI due to the increase in the negative charge on protein.

**FIGURE 3:**
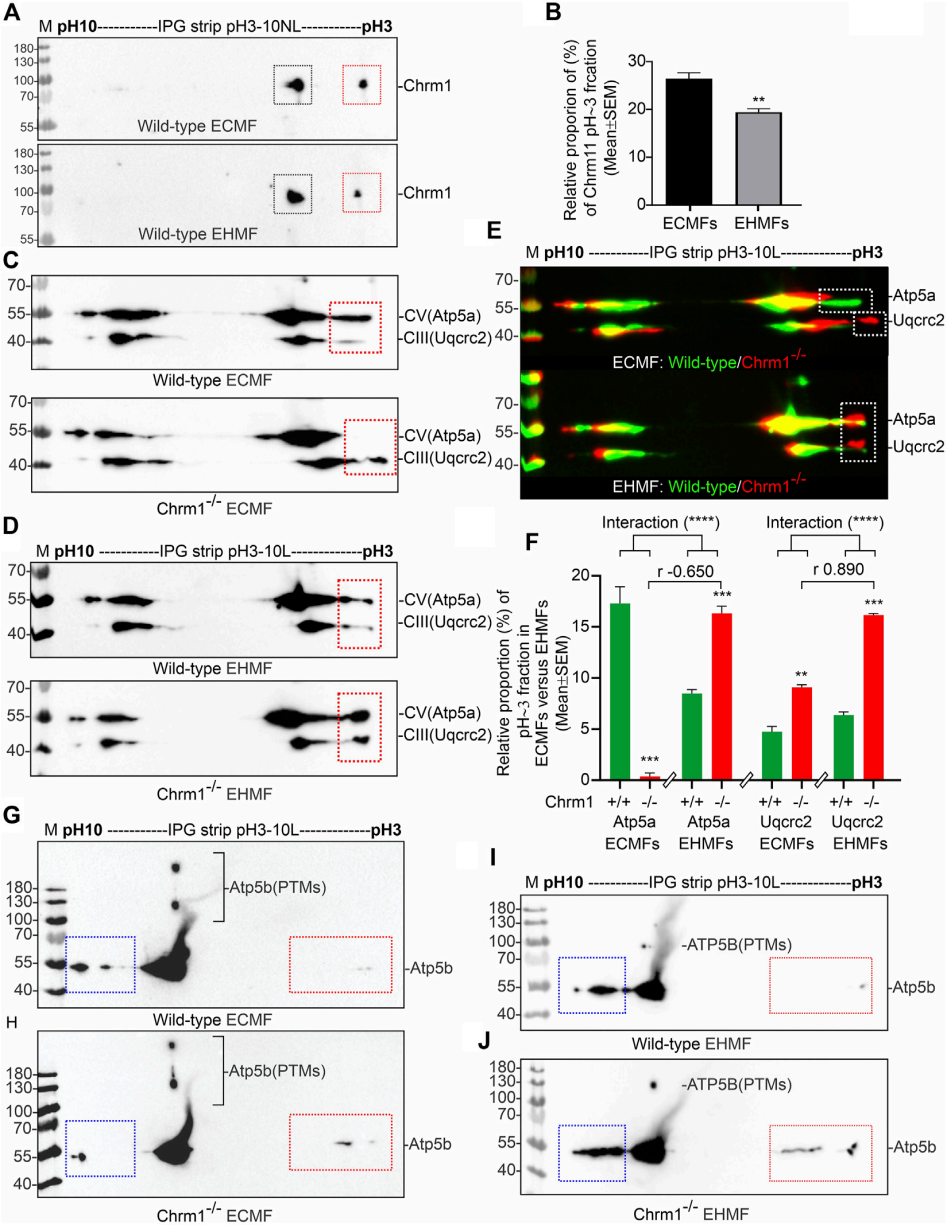
Isoelectric focusing of Chrm1, Atp5a, Atp5b, and Uqcrc2 in EHMFs and ECMFs derived from wild-type and Chrm1^−/−^ mice. **(A)** Immunoblots showing charged fractions of Chrm1 protein in the ECMFs and EHMFs from wild-type mice. **(B)** Bar graphs showing the relative percentage of pH∼3 fraction (red rectangle) of Chrm1 protein in three wild-type ECMFs and EHMFs. Statistical significance by *t*-test (unpaired). (CD): Immunoblots showing charged fractions of Atp5a and Uqcrc2 in ECMFs **(C)** and EHMFs **(D)** derived from wild-type and Chrm1^−/−^ mice. The immunoblots presented in C and D were false-colored and overlaid in E to highlight the region-specific difference in the charged fractions (PTMs) of respective proteins under Chrm1 loss condition. **(E)** The immunoblots presented in C and D were false-colored and overlaid to highlight the tissue-specific difference in PTMs (charged fractions) under Chrm1 deletion condition. **(F)** Bar graphs showing relative percentage of the pH∼3 fractions (white dotted rectangles in E) Atp5a and Uqcrc2 proteins in the ECMFs and EHMFs of six wild-type and six Chrm1^−/−^ mice. The r value indicates Pearson correlation coefficient and the “interaction” *p*-value was determined by an ordinary 2-way ANOVA test. **(G, H)** Immunoblots showing charged fractions (colored dotted rectangles) of Atp5b in the ECMFs of the wild type **(G)** and Chrm1^−/−^ **(H)** mice. **(I, J)** Immunoblots showing charged fractions (colored dotted rectangles) of Atp5b in the EHMFs of the wild type **(I)** and Chrm1^−/−^ **(J)** mice.

First, we performed IEF of Chrm1 protein to see if there is any tissue-specific (hippocampus versus cortex) difference. Muscarinic receptors are phosphorylated during the agonist-induced desensitization process (Waugh et al., 1999). Multiple phosphorylations of Chrm1 in the intracellular loop III region have been previously reported (Butcher et al., 2016). The theoretical pI of Chrm1 protein (transcript ID: ENSMUST00000163785.2) is 9.38. IEF focusing using 3–10 immobilized pH gradient (IPG) followed by SDS-PAGE and immunoblotting using anti-Chrm1 antibody revealed two major charged fractions at pH∼5 and pH∼3 in the ECMFs and EHMFs (Figure 3A, black and red rectangles, respectively). The pH∼3 fraction is the most negatively charged and may represent the maximum phosphorylated fraction which may arise due to the desensitization process during active GPCR signaling. Though, the relative quantification revealed significantly decreased proportion of pH∼3 fraction in the EHMFs compared to ECMFs (Figure 3B), indicating some tissue-specific difference in the rate of phosphorylation, but overall, this indicates active Chrm1 signaling in both cortical and hippocampal tissues.

IEF using nonlinear (NL) immobilized pH 3-10NL IPG gel-strip followed by SDS-PAGE and immunoblotting using a cocktail of anti-OXPHOS antibodies revealed that Atp5a (complex V), Uqcrc2 (complex III), and Sdhb (complex II) proteins were differentially charged in ECMFs versus EHMFs under Chrm1 loss condition (Supplementary Figures S2A–F, colored arrows). The theoretical pI of Atp5a (Transcript ID: ENSMUST00000026495.14), Uqcrc2 (Transcript ID: ENSMUST00000033176.6), and Sdhb (Transcript ID: ENSMUST00000010007.9) are 9.2, 9.2, and 8.9, respectively. All fractions of these proteins in ECMFs appeared as condensed spots on the IPG gel strip within a narrow acidic pH range (pH∼3) (Supplementary Figures S2A, B). A negatively charged fraction of Atp5a (Supplementary Figures S2C, F, red arrows) was more abundant in EHMFs but not in ECMFs under Chrm1 loss condition indicating differential tissue-specific PTMs (potential phosphorylation). The Uqcrc2 PTMs appeared to be different in Chrm1^−/−^ ECMFs versus EHMFs compared to the respective wild-type EHMFs/ECMFs (Supplementary Figures S2C, F, green arrows). The anti-mt-Co1 and anti-Ndufb8 antibodies failed to recognize respective proteins in IEF/SDS-PAGE/immunoblotting (Supplementary Figures S2A–F) compared to SDS-PAGE/immunoblotting (Figure 1I, 2A, B) which is possibly due the difference in the sample preparation that may have masked the epitopes. On the other hand, Sdhb exhibited a difference in the appearance of pH∼3 fractions in Chrm1^−/−^ ECMFs versus EHMFs compared to the respective wild-type. Furthermore, a basic (pH∼7–10) fraction of Atp5a, Uqcrc2, and Sdhb was found relatively less abundant in ECMFs compared to EHMFs (Supplementary Figures S2C, F, yellow dotted rectangles). Overall findings indicate the existence of a considerable tissue-specific difference in the PTMs of OXPHOS-associated proteins under Chrm1 loss.

The close spacing of the observed focused spots in pH3-10 NL IPG strips (Supplementary Figures S2A–E) makes it difficult to interpret some of the PTM data with confidence. Therefore, we used a pH 3–10 linear (L) IPG strip to further resolve the closely spaced charged fractions (spots) of Atp5a and Uqcrc2 in ECMFs (Figure 3C) and EHMFs (Figure 3D). As expected, a pH∼3 (Red dotted rectangles) fraction of both Atp5a and Uqcrc2 exhibited considerable tissue-specific differences in respective tissues (Figure 3E). Quantification based on immunoblots revealed a significant (*p* = 0.0005) reduction of Atp5a pH∼3 fraction in Chrm1^−/−^ ECMFs compared to wild-type, whereas it was significantly (*p* = 0.0006) increased in Chrm1^−/−^ EHMFs compared to wild-type (Figure 3F). On the other hand, the Uqcrc2 pH∼3 fraction was significantly (*p* = 0.0015 and < 0.0001, respectively) increased in both Chrm1^−/−^ ECMFs and EHMFs compared to respective wild-types (Figure 3F). Alteration in the relative proportion of cortical and hippocampal Atp5a pH∼3 fractions under Chrm1 deletion condition were negatively (r = −0.65) correlated, whereas the tissue-specific alteration in Uqcrc2 pH∼3 fractions under Chrm1 deletion condition were positively (r = 0.89) correlated (Figure 3F). Furthermore, we performed a 2-way ANOVA to see if two independent variables, that is Chrm1 deletion status and tissue-specificity have an effect on the post-translational modification (pH3 fraction) of Atp5a and UqCrc2 (dependent variables) proteins. Two-way ANOVA revealed significant interaction (*p* < 0.0001) between the Chrm1 deletion status and tissue-specificity affecting the PTM status of both Atp5a and Uqcrc2 proteins (Figure 3F). Overall, these data indicate that increased PTMs (pH3 fraction) of Atp5a is associated with increased supramolecular assembly leading to MCs formation and enhanced respiration in the hippocampus, whereas, in the cortex, it was found just the opposite, that is decreased PTMs (pH3 fraction) of Atp5a is associated with the decreased supramolecular assembly of Atp5a (MC) and dampened respiration (Table 2) (Sabbir et al., 2023).

**FIGURE 4:**
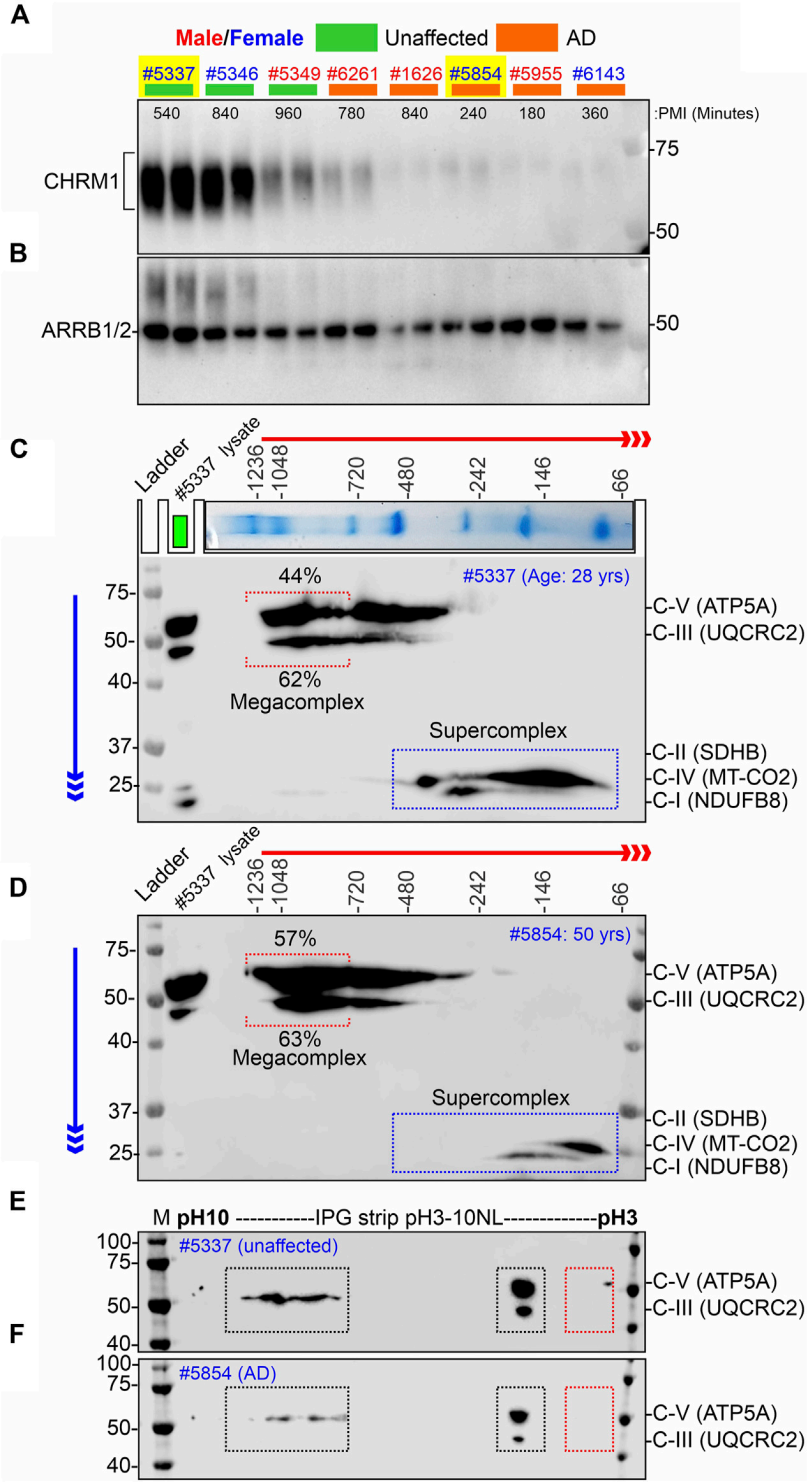
Alzheimer’s hippocampus with severe loss of CHRM1 exhibited a dramatic increase in ATP5A-associated MCs compared to a non-demented individual with a relatively normal hippocampal CHRM1 protein level. **(A, B)** Immunoblots showing the abundance of CHRM1 **(A)** and β-Arrestin 1/2 proteins in non-demented and AD patient-derived hippocampi, postmortem. The yellow highlighted sex-matched samples (Identification numbers: 5337 and 5854) were selected for BN-PAGE and IEF-based analyses. Note the high abundance of CHRM1 in a non-demented individual compared to a low abundance of CHRM1 in an AD patient. **(C, D)** BN-PAGE/SDS-PAGE-based immunoblots showing OXPHOS-associated MPCs in non-demented **(C)** and AD **(D)** hippocampi, postmortem. The immunoblots were generated by the simultaneous use of a cocktail of five antibodies (Table 1). Representative total protein lysates were loaded in the left lane (green rectangle) of the 2D SDS-PAGE to highlight the relative abundance of OXPHOS proteins and their supramolecular assembly. The red and blue arrows indicate the direction of electrophoresis during 1D BN-PAGE and 2D SDS-PAGE, respectively. The red dotted rectangle indicates ≥∼720 kDa Atp5a and Uqcrc2 associated MCs. The relative proportion of these fractions was highlighted below a red bracket. **(E, F)** Immunoblots showing charged fractions of Atp5a and Uqcrc2 in non-demented and AD patients’ hippocampus tissues, postmortem. Colored dotted rectangles represent differently charged fractions.

The catalytic portion of mitochondrial ATP synthase consists of 5 different subunits (*α*, *β*, *γ*, *δ*, and *ε*) assembled with a stoichiometry of 3α, 3β, 1γ, 1δ, and 1ε (Spikes et al., 2020). Subunits alpha (Atp5a) and beta (Atp5b) form the catalytic core. We studied the charged fractions of Atp5b in ECMFs and EHMFs under Chrm1 loss conditions to investigate if PTMs of an Atp5a-adjacent unit in the catalytic core are brain region-specifically altered or not. The theoretical molecular weight and pI of Atp5b (Transcript ID: ENSMUST00000026459.6) are 56 kDa and 5.19 respectively. IEF revealed Atp5b appeared as a major pH∼7-8 fraction and minor basic (pH∼10, blue dotted rectangles) and acidic (pH∼3, red dotted rectangles) fractions (Figures 4G–J). In addition, some high molecular weight fraction of Atp5b was also observed (Figures 4G–J). Interestingly, the basic fraction of Atp5b appeared relatively less abundant in ECMFs compared to EHMFs (Figures 4G–J, blue rectangles), indicating fundamental brain region-specific differences exist. However, loss of Chrm1 in the hippocampus versus cortex exhibited no effect on Atp5b PTMs. Thus, overall findings indicate that Chrm1 loss selectively affected only Atp5a, but not the adjacent protein Atp5b in the catalytic core of ATP synthase.

### 3.6 A pilot study involving a hippocampus from the postmortem brain of an AD patient with that of a non-AD afflicted individual revealed a greater assembly of ATP5A (complex-V)-associated MCs in the AD hippocampus, whereas assembly of MT-CO_2_ (complex-IV) and NDUFB8 (complex-I) associated SCs were reduced in the AD hippocampus compared to a non-AD individual

Our retrospective analysis using a large cohort (AD: N = 74, non-demented: N = 19) of post-mortem hippocampus tissues revealed that hippocampal loss of CHRM1 is not associated with patient survival. Interestingly, in this study, we demonstrated that loss of Chrm1 in mouse hippocampus improved mitochondrial function that was associated with increased MC assembly and PTMs (pH∼3 fractions) of OXPHOS associated proteins, specifically Atp5a and Uqcrc2 proteins (Figures 2, 3). Based on this, we hypothesized that CHRM1 deficient AD patients would exhibit enhanced supramolecular assembly and PTMs of ATP5A and UQCRC2 proteins. This hypothesis was tested as a proof-of-concept by comparing the 2D BN-PAGE/SDS-PAGE-based SC/MC profile and IEF/SDS-PAGE-based PTMs (charged fractions) profile of the OXPHOS-associated proteins, specifically ATP5A and UQCRC2 in the *post mortem* hippocampus of a non-demented individual with relatively normal CHRM1 protein, to an AD patient with severely reduced hippocampal CHRM1 protein (Fig 4AB). As anticipated BN-PAGE revealed relatively increased (57% versus 44%) ATP5A MCs (≥720 kDa) but relatively unaltered (63% versus 62%) UQCRC2 MCs (≥720 kDa) in CHRM1 reduced AD hippocampus compared to the CHRM1 positive non-demented individual (Figures 4C, D, red dotted brackets). In contrast to the improved ATP5A MCs in CHRM1 reduced AD hippocampus, the SCs associated with MT-CO2 (complex IV) and NDUFB8 (complex I) were shifted to relatively lower molecular weight regions (100–480 kDa versus 66–146 kDa) (Figures 4C, D, blue dotted rectangles) indicating loss of associated interacting proteins. These findings support our hypothesis that loss of CHRM1 in the human hippocampus enhances the supramolecular assembly of ATP5A, which may have functional benefits that would not be disadvantageous from the survival point of view.

IEF revealed both ATP5A and UQCRC2 appeared as basic (pH∼7–10) and acidic (pH∼3–4) fractions in postmortem human hippocampus tissues (Fig 4EF) as previously observed in mice (Figure 3; Supplementary Figure S2). However, the extremely negative fraction (pH∼3) of these proteins was not observed in human tissue samples. The significantly increased pH∼3 fraction of Atp5a and Uqcrc2 proteins in Chrm1^−/−^ mouse hippocampus was associated with enhanced MC assembly of these proteins. This deviation between mouse and human hippocampus-based studies may be due to the postmortem delay of tissue harvesting, 4- and 9-h intervals after death, during which time the altered physiological status of the brain cells may affect the PTMs and therefore, is not reflective of the PTMs of freshly harvested mouse tissues. This argument is supported by the reports that post-mortem interval affects the phosphorylation (negatively charged fraction) of signaling proteins (Li et al., 2003; Wang et al., 2015).

### 3.7 In contrast to the cortical effects of Chrm1 deletion, hippocampal Chrm1 deletion caused no discernible effect on the tinctorial property of pyramidal neurons nor did it adversely affect mitochondrial ultrastructure

We analyzed the distribution and ultrastructure of pyramidal neurons in the hippocampus by TEM to understand the impact of Chrm1 loss. Toluidine-stained semithin (∼500 nm thick) sections (Supplementary Figure S3) and TEM images (S4AB) revealed the presence of 2 types of neuronal populations; light and dark neurons. In contrast to the cortex where dark neurons are less frequent but whose occurrence increases significantly with Chrm1 loss condition (Table 2) (Sabbir et al., 2023), the hippocampus exhibited predominantly dark pyramidal neurons (98% in the wild-type), with their frequency remaining unaltered in the Chrm1 knockout hippocampus (Supplementary Figures S3C, D), indicating a brain region-specific effect (Table 2).

**FIGURE 5:**
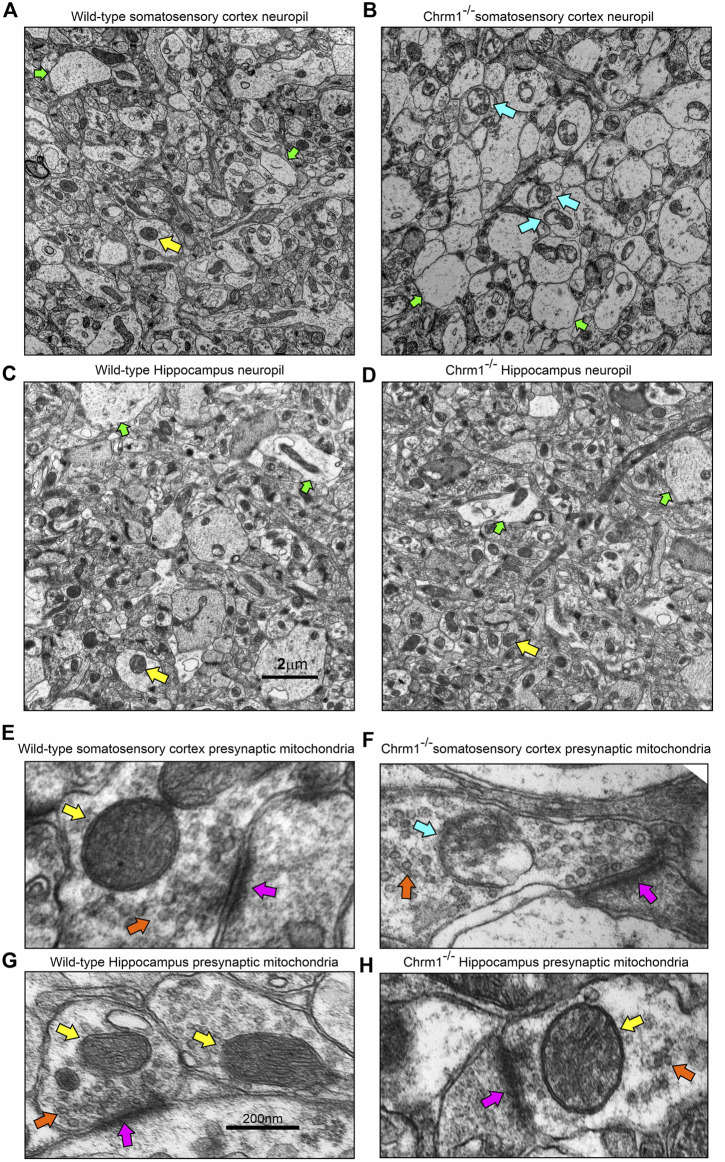
Loss of Chrm1 in the hippocampus exhibited no effect on neuropil mitochondrial ultrastructure, whereas cortical loss exhibited abnormal mitochondria in the neuropil synaptic buds. **(A–D)** TEM images of cortical neuropil in wild-type **(A)** and Chrm1^−/−^ **(B)** mice. **(C, D)** TEM images of hippocampal neuropil in wild-type **(A)** and Chrm1^−/−^ **(B)** mice. The yellow arrows: are normal mitochondria, the green arrows are less electron-dense post-synaptic terminals, and blue arrows are abnormal mitochondria. **(E, F)** A magnified image of the representative wild-type **(E, G)** and Chrm1^−/−^ **(F, H)** Somatosensory cortex and hippocampal synaptic terminals showing mitochondrial ultrastructure. The pink arrows are synapse, the orange arrows are synaptic vesicles, yellow arrows are normal mitochondria, and the blue arrow is an abnormal mitochondrion.

Furthermore, TEM-based images revealed no mitochondrial ultrastructural defect in Chrm1^−/−^ hippocampal dark neurons compared to wild-type (Supplementary Figure S4, orange arrows), in contrast to the damaging effect of Chrm1 deletion on cortical mitochondrial ultrastructure in the dark neurons (Table 2) (Sabbir et al., 2023). On the other hand, the ER structures are less visible or the cisternae of the ER are relatively narrower in the Chrm1^−/−^ hippocampal dark neurons compared to wild-type, a phenotype that was opposite to cortical neurons with deleted Chrm1 (Table 2) (Sabbir et al., 2023). Similarly, in contrast to the presence of abnormal mitochondria (Figures 5A, B, E, F, yellow versus blue arrows) and increased frequency of less electron-dense synaptic nerve terminals (Figures 5A, B, green arrows) in the Chrm1^−/−^ cortical neuropil compared to wild-type, Chrm1 deletion caused no abnormalities of mitochondrial ultrastructure in the hippocampus (Figures 5C, D, yellow arrows), nor any alteration of the tinctorial properties of its synaptic nerve terminals Figures 5C, D, green arrows. The less electron-dense synapse (Figures 5E–H, pink arrows) terminals were possibly post-synaptic due to a lack of identifiable synaptic vesicle-like structures (Figures 5E–H, orange arrows). Overall, these findings indicate that the alteration in the tinctorial property of the pyramidal neurons and mitochondrial ultrastructural abnormalities under Chrm1 deletion condition was brain region-specific, with the latter being associated with respiratory function.

## 4 Discussion

**FIGURE 6:**
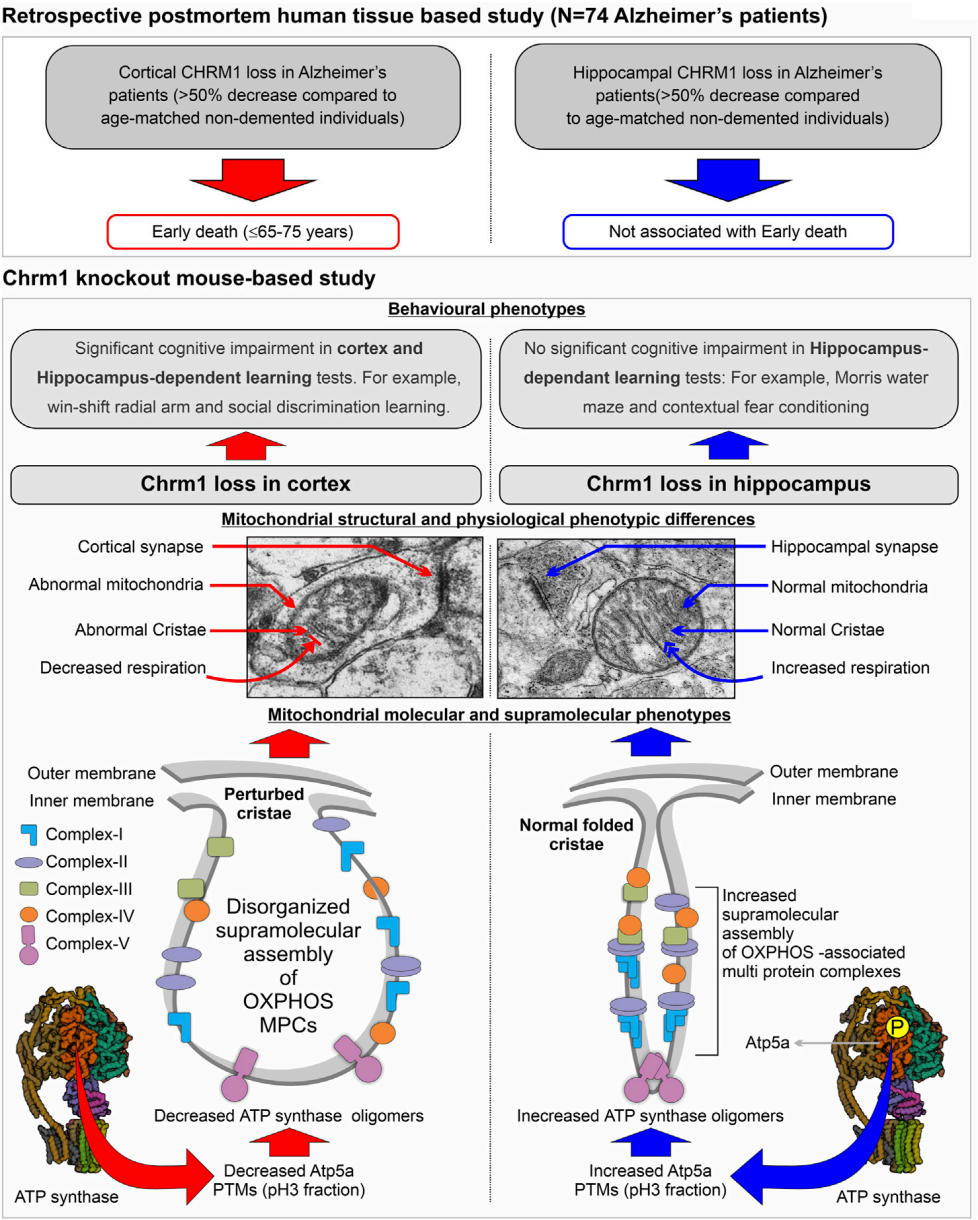
Model summarizing the differential effect of the deletion of Chrm1 on mouse hippocampus versus cortical mitochondrial molecular, ultrastructural, and physiological phenotypes: Inferences for Chrm1-deficient mouse neuropathologies and behavioral anomalies to represent the difference in the survival of Alzheimer’s patients with cortical versus hippocampus loss of CHRM1.

An overview of the mouse hippocampus versus cortex-specific differential effect of Chrm1 deletion on mitochondrial phenotypes, its implications in understanding the relationship between hippocampal versus cortical loss of CHRM1 in AD patients and disease outcome (survival), and the mechanistic interpretation of some behavioral phenotypes observed in Chrm1^−/−^ mice have been diagrammatically summarized in Figure 6. In the present study, using targeted Chrm1 deleted mice, we demonstrated that the loss of Chrm1 in the hippocampus led to an enhancement of the supramolecular assembly of OXPHOS-associated proteins, specifically Atp5a, mt-Co1, Uqcrc2, and Sdhb subunits representing mitochondrial complexes II–V, respectively (Figure 2). The enhanced supramolecular arrangement of complexes II-V may explain the improved respiratory function observed in Chrm1^−/−^ EHMFs compared to wild-type EHMFs (Figure 1). Furthermore, in contrast to the cortex, the deletion of Chrm1 did not alter mitochondrial abundance or ultrastructure in the hippocampal neuropil (Figure 5). The most striking observation is the difference in PTMs of hippocampus versus cortical OXPHOS-associated proteins, specifically Atp5a, Uqcrc2, and Sdhb. More importantly, deletion of Chrm1 brain region-specifically altered multiple Atp5a-associated molecular phenotypes that are potentially inter-linked. These brain region-specific differential effects of Chrm1 loss can be sequentially arranged in the following order to interpret our results. In the cortex: decreased Atp5a phosphorylation (pH∼3 fractions) → decreased Atp5a MCs → ultrastructural abnormalities in the cristae → reduced respiration. Whereas in the hippocampus: increased Atp5a phosphorylation (ph3 fraction) → increased Atp5a MCs → normal cristae → enhanced respiration. Thus, the loss of Chrm1 signaling in the hippocampus may be beneficial while the loss of Chrm1 in the cortex is degradative. This could provide a mechanistic explanation of the retrospective postmortem human tissue-based observation that reduced cortical CHRM1 in a subset of AD patients is positively correlated to poor survival, i.e., an earlier age of death, whereas hippocampal CHRM1 is not linked to an earlier age of death. Furthermore, the effect of Chrm1 loss in mouse hippocampus leading to enhanced Atp5A MCs was recapitulated in a pair of post-mortem human hippocampi comparing an AD hippocampus with reduced CHRM1 expression to that of a non-demented individual with normal hippocampal CHRM1 expression (Figure 4). This suggests a functional relevance of this Chrm1^−/−^ mouse-model-based findings to understand the pathogenesis and outcome of AD. Additionally, the unique observations in this study may help us understand some previously unexplained behavioral phenotypes observed in Chrm1 knockout mice that are discussed in subsequent sections.

One major observation in this study is the establishment of a cause-and-effect relationship between Chrm1 signaling with the PTMs and supramolecular assembly of the Atp5a subunit (representing Complex V) underlying mitochondrial structure and function *in vivo*. In the hippocampus, we showed that loss of Chrm1 led to an enhancement of ≥ 720 kDa Atp5a MCs associated with increased potentially phosphorylated PTMs (pH∼3 fractions) of Atp5a (Figures 3, 4), improved respiration (Figure 1), and no mitochondria structural deficits (Figures 6, 7). On the other hand, our previous study (Sabbir et al., 2023) demonstrated that loss of Chrm1 in the cortex led to a reduced ≥720 kDa Atp5A MCs which was associated with dampened respiration (Figure 1), severe structural deficits in the mitochondria (Figures 6, 7) and decreased PTMs (pH∼3 fractions) of Atp5a (Figures 3, 4). Previously, we demonstrated that overexpression of Chrm1 in transformed cells that do not express native Chrm1 protein caused an enhancement of the ≥ 720 kDa Atp5A MCs (Sabbir et al., 2023). Taking these observations together, it can be suggested that Chrm1 signaling regulates PTMs of Atp5a which may be responsible for the ATP synthase MC assembly, that in turn may regulate mitochondrial cristae structure and function. Atp5a is a component of the catalytic core (F1) of the mitochondrial ATP synthase enzyme complex (Jonckheere et al., 2012). The link between ATP synthase MCs and mitochondrial ultrastructure has been reported in the literature. Cryoelectron microscopy reveals that ATP synthase dimers shape the extensively folded inner membrane of mitochondria (Guo et al., 2017b). Long rows of dimers, interacting to form ATP synthase MCs, force the membrane to maintain its convexity at the apex of the cristae, thereby regulating mitochondrial ultrastructure (Nesci and Pagliarani, 2019). Cristae shape determines mitochondrial respiratory efficiency (Cogliati et al., 2013). Therefore, it is reasonable to conclude that decreased Atp5a MCs in the Chrm1^−/−^ cortex deform mitochondrial cristate structure, decreasing respiration rate, whereas increased Atp5a MCs in the Chrm1^−/−^ hippocampus prevent structural deterioration of cristae, maintaining or even enhancing respiration. Future in-depth studies are required to address the next important question: How PTMs of Atp5a regulate MC assembly, and how Chrm1 regulates Atp5a PTMs?

The brain region-specific increase in the hippocampus and decrease in the cortex of Atp5a PTMs (pH 3 fraction) in Chrm1^−/−^ mouse brains was associated with a corresponding increase or decrease of its MC assembly, indicating a link between PTMs and MC assembly (Sabbir et al., 2023). The role of PTMs in the ratio of MC to SC assembly or function has not been well studied in the past, but there is a large body of information on PTMs of individual respiratory complex subunits and other mitochondrial proteins. For example, the Phosphositeplus database (Hornbeck et al., 2015) has archived a variety of PTMs, including phosphorylation and acetylation of OXPHOS proteins. It has been suggested that mitochondrial SC stability and function can be altered by phosphorylation/dephosphorylation or acetylation/deacetylation of its component subunits (Acin-Perez and Enriquez, 2014). Several studies have shown that kinase cascades and protein phosphorylation control mitochondrial function (Gibson, 2005; Horbinski and Chu, 2005; Huttemann et al., 2007). Chrm1 is coupled to or intersects with, signaling pathways that involve sequential activation of serine/threonine protein kinases, specifically the mitogen-activated protein kinase (MAPK) pathway (Hamilton and Nathanson, 2001; Sabbir and Fernyhough, 2018). MAPKs, directly and indirectly, target mitochondrial proteins regulating their function (Javadov et al., 2014), therefore, it is tempting to suggest that Chrm1-MAPK signaling or yet unidentified Chrm1 downstream kinases may phosphorylate Atp5a. Until recently, there are few reports about the phosphorylation of ATP synthase subunits attributable to specific kinase functions (Bernardi et al., 2015). Phosphorylation of Atp5b has been linked to increased dimerization (Kane et al., 2010), while we have demonstrated that Atp5b is differentially modified (charged fractions) in a brain region-specific manner (Figure 3). Mass spectrometric analysis of Atp5a in the rat brain mitochondria reveals tyrosine phosphorylation (Augereau et al., 2005; Lewandrowski et al., 2008). Phosphorylation of Atp5a at S184 and S419 residues has also been reported (Huttlin et al., 2010). Differential phosphorylation of different components of the ATP synthase in failing versus normal hearts has been reported (Kane and Van Eyk, 2009; Bernardi et al., 2015; Stram and Payne, 2016). This information suggests that an in-depth mass spectrometry-based analysis using an immunoprecipitated fraction of OXPHOS proteins from wild-type and Chrm1^−/−^ brain region samples could pinpoint specific modifications, unraveling their functional relevance. Overall, the loss of Chrm1 signaling mediated brain region-specific alterations of the OXPHOS proteome and its supramolecular assembly indicates functional relevance in neurodegenerative disease.

Another important question is: what is the link between the brain region-specific effect of Chrm1 loss on mitochondrial physiology and cognitive function? Neurons critically depend on mitochondrial function for neurotransmission and plasticity (Kann and Kovács, 2007). Our findings indicate that hippocampal loss of Chrm1 is not detrimental to mitochondrial function, but cortical loss severely affected structural, molecular, and physiological aspects of mitochondrial function. This implies that hippocampal loss of Chrm1 may not lead to cognitive decline, but a cortical loss of Chrm1 may harm cognitive function. This concept is supported by behavioral studies using Chrm1^−/−^ mice. To determine whether deletion of Chrm1 leads to the impairment of cognitive processes, Miyakawa et al. (2001) subjected Chrm1^−/−^ mice to several hippocampus-dependent learning and memory tasks. Surprisingly, Chrm1^−/−^ mice displayed no significant cognitive impairments in contextual fear conditioning or the Morris water maze, a test that is frequently used to assess spatial reference memory in rodents (Miyakawa et al., 2001). Chrm1^−/−^ mice were hyperactive, with the degree of hyperactivity correlating with performance deficits in different behavioral tests (Miyakawa et al., 2001). Interestingly, a related study by Anagnostaras et al. showed that Chrm1^−/−^ mice displayed normal and enhanced memory for tasks that involved matching-to-sample problems (Morris water maze and contextual fear conditioning), but they were severely impaired in non-matching-to-sample working memory as well as consolidation (win-shift radial arm and social discrimination learning) (Anagnostaras et al., 2003). Anagnostaras et al. (2003) proposed that Chrm1 is specifically involved in the memory process for which the cortex and hippocampus interact. Consolidation is a process by which the hippocampus guides the reorganization of the information that is stored in the neocortex such that it eventually becomes independent of the hippocampus (Preston and Eichenbaum, 2013; Squire et al., 2015). Both consolidation and non-matching-to-sample working memory require memory circuits in the cortex (Kowalska et al., 1991; Meunier et al., 1997; Buffalo et al., 1999). As noted above, resting cortical neuron consume approximately 4.7 billion molecules of ATP per second (Zhu et al., 2012), with the majority of this energy being used for synaptic neurotransmission. Therefore, structurally abnormal, functionally impaired mitochondria at Chrm1^−/−^ cortical synapses may not be capable of meeting the energy demand to maintain synaptic transmission needed for the memory consolidation process, thereby accounting for the cortex-dependent behavioral abnormalities in the Chrm1^−/−^ mice (Figure 6). On the other hand, normal to enhanced mitochondrial function in the hippocampus of Chrm1^−/−^ mice may improve hippocampal dependent learning and memory tasks (Miyakawa et al., 2001). Overall, this interpretation of hippocampal versus cortical response to the deletion of Chrm1, and its differential effect on their mitochondrial and cognitive functions supports our recent retrospective postmortem human tissue-based observations showing that hippocampal loss of CHRM1 in a subset of AD patients is not associated with poor survival, but that cortical CHRM1 loss is strongly associated with poor disease outcome (early death ≤65–75 years) (Sabbir et al., 2022).

Loss of Chrm1 differentially affected the distribution of pyramidal neuronal cell types in the cortex and hippocampus which was characterized by the tinctorial properties of the neurons based on toluidine staining or heavy metal contrast staining (uranyl and lead acetate) in electron microscopy. Previously we showed that dark neurons are very infrequent in the cortex and Chrm1 loss resulted in a significantly increased number of cortical dark neurons compared to wild-type (wild-type versus Chrm1^−/−^: 2% versus 82%). In this study, we showed that dark neurons are predominant in hippocamps (Supplementary Figure S3). Approximately 98% of the pyramidal neurons in the CA1 region are dark in the wild-type. In contrast to the cortex (Sabbir et al., 2023), the frequency of dark neurons in Chrm1^−/−^ hippocampus was not altered (Supplementary Figures S3, S4). The biochemical properties of dark neurons are reviewed in detail by Sabbir et al. (2023). It has been demonstrated that treatment with pharmacological agents targeted to N-methyl-D-aspartate (NMDA) and non-NMDA receptors abolished dark neurons in rat cortex (Kherani and Auer, 2008). This suggested a role for the pharmacologic subtypes of glutamate receptors in the pathogenetic mechanism of dark neuron formation (Kherani and Auer, 2008). Therefore, it can be suggested that dark neurons may be glutamatergic, and their frequency was dramatically increased in the Chrm1 deficient cortex, but not in the hippocampus. Glutamatergic neurons are primarily excitatory and their prolonged action leads to excitotoxicity that is known to play a role in the pathophysiology of a variety of diseases such as epilepsy or AD (Jewett and Thapa, 2022). Prolonged activation of glutamate receptors starts a cascade of neurotoxicity that ultimately leads to the loss of neuronal function and cell death (Armada-Moreira et al., 2020). Interestingly, the glutamatergic pyramidal neurons account for ∼90% of hippocampal neurons, a much higher percentage than in other parts of the cortex, supporting our observations of dark neuron frequencies in the hippocampus (Olbrich and Braak, 1985). Further support for this hypothesis comes from the observation that ACh can modulate glutamatergic inputs to pyramidal neurons in the amygdala by presynaptic as well as postsynaptic Chrm1-mediated mechanisms (McDonald et al., 2019). On the other hand, Chrm4, a paralog of Chrm1, mediates the cholinergic inhibition of glutamatergic response in corticostriatal neurons (Pancani et al., 2014). Activation of presynaptic Chrm4 receptors leads to a decrease in glutamate release from corticostriatal terminals (Pancani et al., 2014). These observations indicate some tissue-specific link between Chrm1 and glutamatergic signaling underlying AD pathogenesis. More in-depth future studies are warranted in the future to understand the mechanistic basis of Chrm1 loss and differential tissue-specific effects in altering the biochemical and ultrastructural properties of glutamatergic neurons in the brain.

This study provided novel insight into the hitherto unknown brain tissue-specific role of Chrm1 loss that exhibited an opposing effect on multiple mitochondrial inter-related molecular, physiological, and ultrastructural properties that are highly relevant in understanding the molecular basis of cholinergic hypofunction in Alzheimer’s pathogenesis and disease outcome (Clader and Wang, 2005; Jiang et al., 2014) as well as explaining the cognitive deficits/enhancement in different behavioral studies of Chrm1^−/−^ mice (Miyakawa et al., 2001; Anagnostaras et al., 2003). Our identification of Chrm1 signaling loss in the cortex but not in the hippocampus, as a causal factor leading to mitochondrial structural and functional abnormalities, will set the direction for future Alzheimer’s research. Detailed high throughput mass spectrometric characterization of tissue-specific mitochondrial phosphoproteomic changes under Chrm1 loss of function condition is warranted as an immediate next step to move this field of science forward.

## Data Availability

The original contributions presented in the study are included in the article/Supplementary Material, further inquiries can be directed to the corresponding author.

## Supplementary material

SUPPLEMENTARY FIGURE S1 Ultrastructural analysis of EHMFs.**(A)** TEM images showing the ultrastructure of EHMF derived from wild-type hippocampus. **(B)**: TEM images showing a magnified view of the EHMF showing pre- (green arrows) and post-synaptic (orange arrows) terminals in the enriched fractions containing mitochondria (yellow arrows). The blue arrows are isolated unencapsulated mitochondria. Pink arrows are mitochondria in presynaptic vesicles.

SUPPLEMENTARY FIGURE S2 Isoelectric focusing of OXPHOS proteins in EHMFs and ECMFs derived from wild-type and Chrm1^−/−^ mice. **(A, B, D, E)**: Immunoblots showing charged fractions of OXPHOS proteins in the ECMFs (AB) and EHMFs (DE) derived from wild-type and Chrm1^−/−^ mice. Colored arrows indicate a difference in the PTMs of respective proteins between wild-type and Chrm1^−/−^ samples. **(C, F)** The immunoblots presented in AB and DE were false-colored and overlaid to highlight the tissue-specific difference in PTMs (charged fractions) under Chrm1 deletion condition. The dotted rectangles in C and F highlight the tissue-specific difference in OXPHOS charged fractions.

SUPPLEMENTARY FIGURE S3 Loss of Chrm1 in the hippocampus exhibited no effect on the tinctorial property of the hippocampal pyramidal neurons. **(A, B)**: Toluidine-stained ultrathin sections of the wild-type **(A)** and Chrm1^−/−^ **(B)** mice hippocampi showing different subfields, namely Cornu Ammonis (CA) 1, CA2, CA3, and dentate gyrus (DG). Black rectangles represent CA1 regions shown at higher magnification in C and D. **(C, D)**: Toluidine-stained ultrathin sections showing the frequency of dark (pink arrows) and light (yellow arrows) neurons in the CA1 region of two wild-type **(C)** and Chrm1^−/−^ **(D)** mice hippocampi. Note the frequency of dark neurons was not altered (visually) under the Chrm1 loss condition.

SUPPLEMENTARY FIGURE S4 Deletion of Chrm1 in the hippocampus exhibited no effect on the mitochondrial ultrastructure but altered the ER cisterns in the hippocampal pyramidal neurons. **(A, B)**: Toluidine-stained ultrathin section of a wild-type CA1 subfield of the hippocampus **(A)**. The yellow rectangle marked area represents a region used in TEM analysis to study the ultrastructure of dark neurons **(B)**. The pink arrows are dark (electron-dense) pyramidal neurons and the green arrows are light (less electron-dense) pyramidal neurons. **(C, D)**: TEM images showing the ultrastructure of dark pyramidal neurons. The orange arrows are mitochondria and the cyan arrows are ER. Note the cisterns in the Chrm1^−/−^ dark neurons are narrower and less frequent compared to wild-type.

## Abbreviations

AD, Alzheimer’s; BN-PAGE, Blue-Native Polyacrylamide Gel Electrophoresis; CNS and PNS, central and peripheral nervous systems; ECMFs, Enriched cortical mitochondrial fractions; EHMFs, enriched hippocampal mitochondrial fractions; GPCRs, G-protein-coupled receptors; HD, Huntington’s disease; IEF, Isoelectric focusing; MC, Megacomplex; MPC, multiprotein complex; OCR, oxygen consumption rate; OXPHOS, oxidative phosphorylation; PD, Parkinson’s disease; PTMs, post-translational modifications; SEM, standard error of the mean; SC, Supercomplex; TEM, transmission electron microscopy.
