# Supplementary figures and images for: Hippocampal versus cortical deletion of cholinergic receptor muscarinic 1 in mice differentially affects post-translational modifications and supramolecular assembly of respiratory chain-associated proteins, mitochondrial ultrastructure, and respiration: implications in Alzheimer’s disease
Source: Front Cell Dev Biol. 2023 May 24;11:1179252. doi: 10.3389/fcell.2023.1179252 (PMC10246746; doi:10.3389/fcell.2023.1179252)

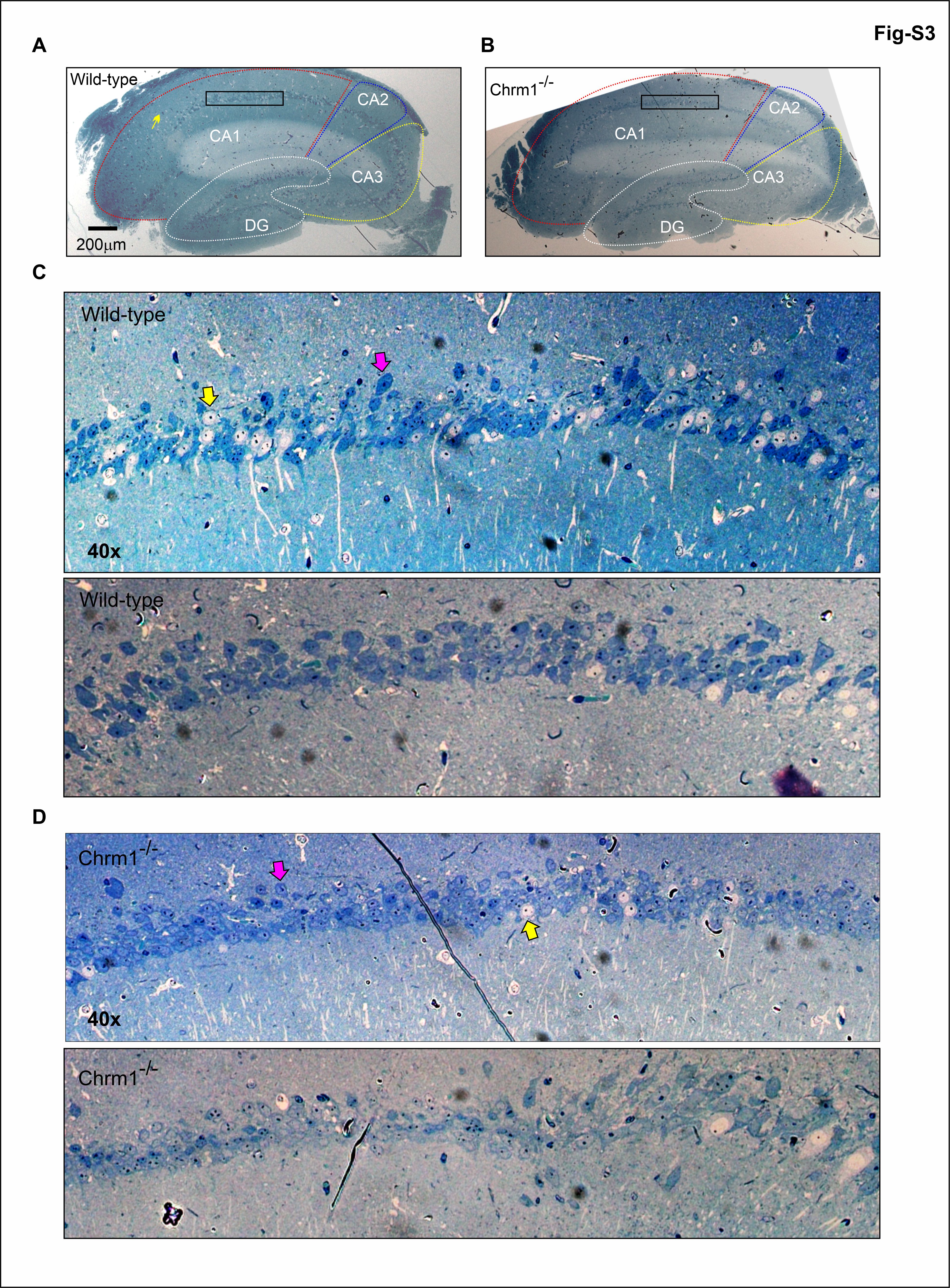

Supplement: Supplementary file 1 [file Image3.JPEG]

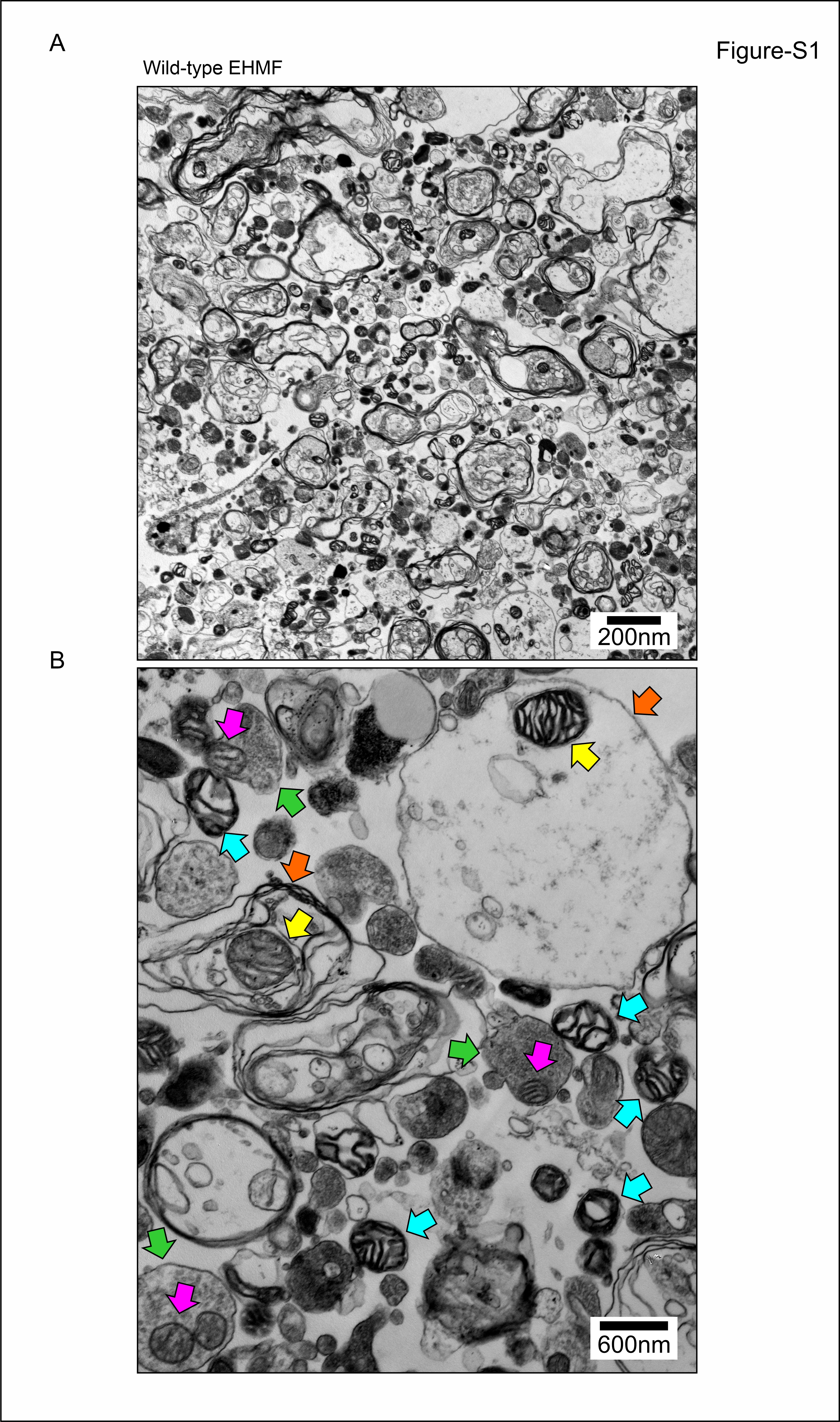

Supplement: Supplementary file 2 [file Image1.JPEG]

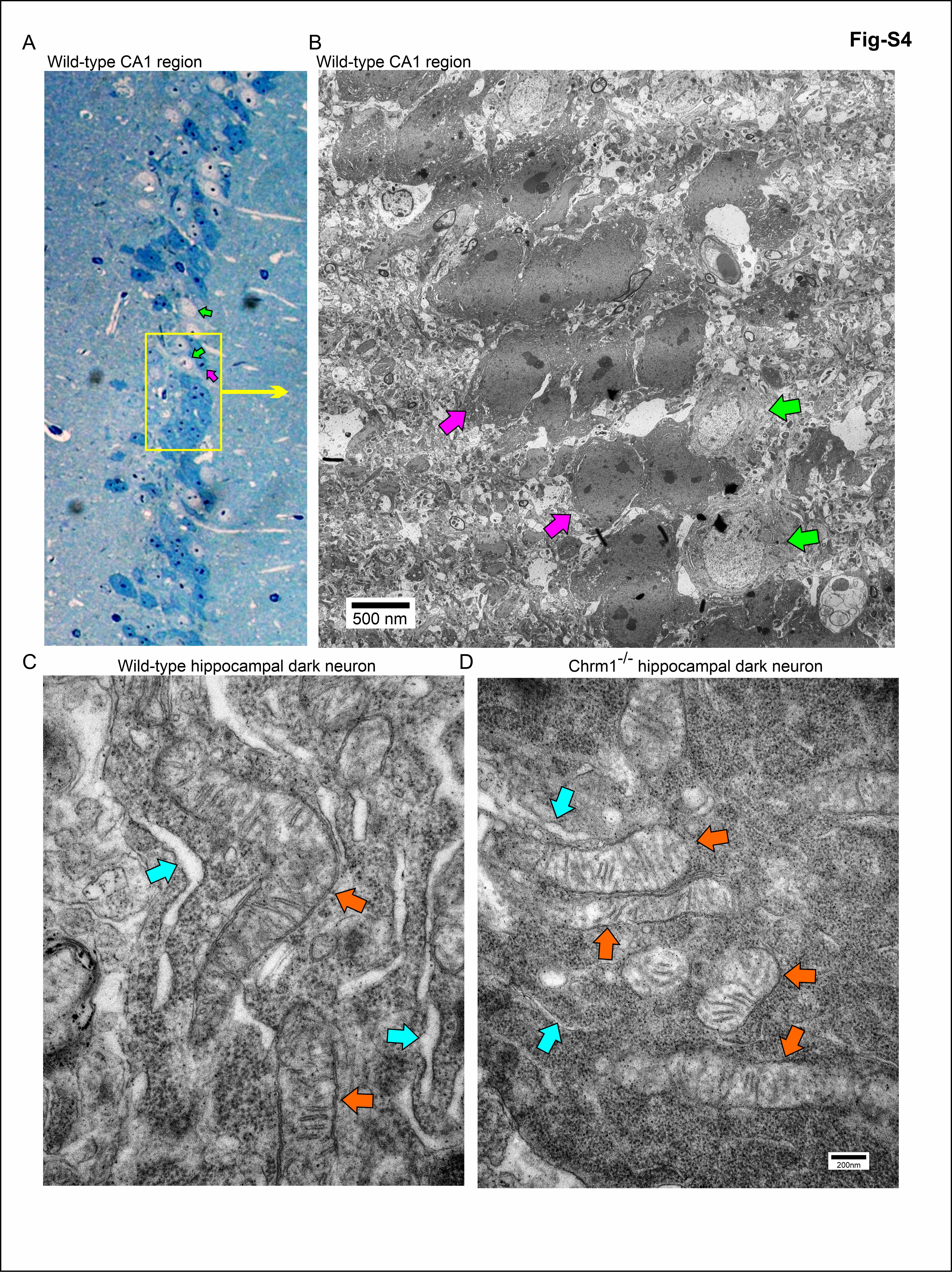

Supplement: Supplementary file 3 [file Image4.JPEG]

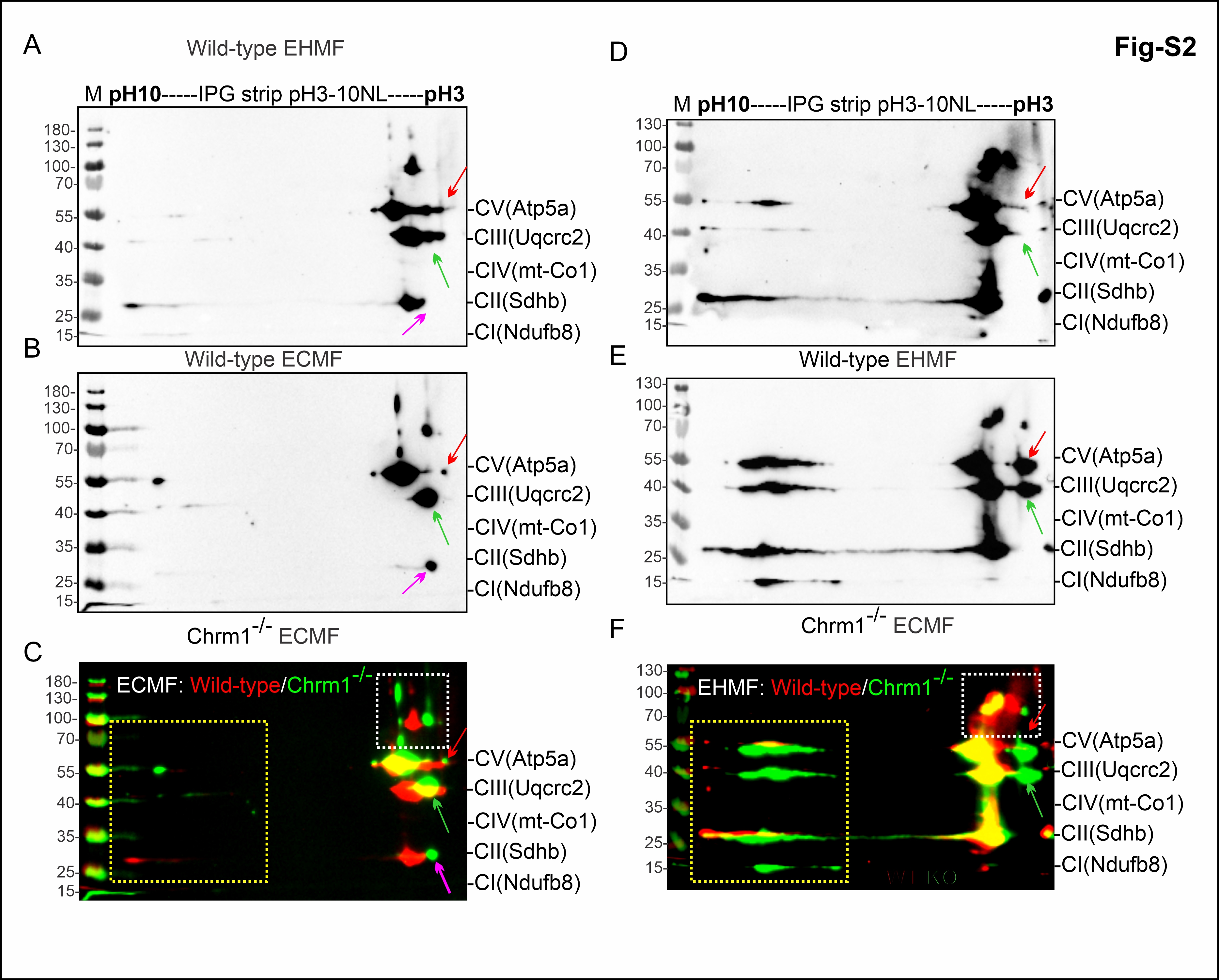

Supplement: Supplementary file 4 [file Image2.JPEG]
